# Potential Therapeutic Effects of Epithelial and Mesenchymal Stem Cell Secretome in Benzalkonium Chloride-Induced Limbal Stem Cell Dysfunction

**DOI:** 10.3390/cells14221790

**Published:** 2025-11-14

**Authors:** Agnieszka Prusek-Kucharek, Bartosz Sikora, Piotr Czekaj

**Affiliations:** Department of Cytophysiology, Chair of Histology and Embryology, Faculty of Medical Sciences in Katowice, Medical University of Silesia in Katowice, 40-055 Katowice, Poland; agnieszka.prusek@sum.edu.pl (A.P.-K.);

**Keywords:** limbal stem cells (LSCs), human adipose-derived stem cells (hADSCs), human amniotic epithelial cells (hAECs), human amniotic mesenchymal stromal cells (hAMSCs), human amniotic cells (hACs), benzalkonium chloride (BAC), conditioned medium (CM)

## Abstract

**Highlights:**

**What are the main conclusions?**

- Conditioned media (CM) derived from adipose tissue-derived stem cells (hADSCs), amniotic epithelial cells (hAECs), and amniotic cells expressing epithelial and mesenchymal markers (hACs) promote the viability of limbal stem cells (LSCs) damaged by benzalkonium chloride (BAC), a commonly used eye drop.
- The effects of these CM administered concurrently with BAC (model 1) or after prior tissue damage with BAC (model 2) differ in terms of modulating inflammation, apoptosis, cell cycle, proliferation, and migration of LSCs.

**What are the implications of the main conclusions?**

- All CM tested neutralize the toxic effects of BAC, but hADSC-CM demonstrates the highest efficacy.
- Administration of CM concurrently with BAC exposure has greater therapeutic potential than the treatment used after toxic injury.
- These findings highlight the promising role of acellular stem cell-based therapies in ophthalmic diseases.

**Abstract:**

Dry Eye Disease (DED) is a multifactorial condition of the ocular surface, with one potential cause being damage from eye drops containing preservatives such as benzalkonium chloride (BAC). Current treatments for DED are unsatisfactory; therefore, it is worth exploring new therapies based on the secretome derived from stem cells. Human stem cells are important sources of growth factors and cytokines that promote tissue regeneration. The secretome of these cells can be obtained in vitro in conditioned medium (CM). The aim of the study was to evaluate the effect of CM derived from adipose-derived stem cells (hADSCs) and amniotic membrane-derived cells expressing mesenchymal and/or epithelial markers on limbal stem cells (LSCs) damaged by BAC, focusing on their regenerative potential. The study used two experimental models: the first focused on neutralizing the toxic effects of BAC when each CM was administered concurrently, and the second on the therapeutic effects of CM after prior cell damage by BAC. The effects of CM on LSCs were assessed, including apoptosis, cell cycle progression, proliferation, migration, and inflammation. CM from ADSCs and amniotic cells were shown to significantly reduce BAC-induced damage to LSCs. All tested CM promoted LSC regeneration, although their efficacy varied among treatments. The application of CM during BAC exposure yielded stronger and more consistent benefits than post-injury treatment.

## 1. Introduction

One of the challenges in treating ocular surface diseases is repairing damage caused by preservatives commonly found in eye drops, such as benzalkonium chloride (BAC) [1]. These agents can be toxic to limbal stem cells (LSCs) and contribute to the development of dry eye disease (DED) [2]. Chemically damaged corneas have been shown to lose transparency, exhibit neovascularization, and display increased levels of inflammatory and proangiogenic markers such as VEGF, MMP-2, MCP-1, IL-6, TNF-α, and TGF-β [3]. New therapeutic opportunities may arise from treatments using stem cells and their secretions, which contain substances that inhibit inflammation or promote tissue regeneration. In vitro, this secretion can be collected from cultures of various types of stem cells as conditioned medium (CM) [4]. Because of potential differences in the composition and efficacy of the secretome derived from phenotypically differentiating cells, it is important to characterize the marker expression profile of the cells from which the conditioned medium is collected. This is particularly relevant for stem cells undergoing epithelial-to-mesenchymal transition (EMT) or expressing varying markers at different stages of differentiation [5].

Adipose tissue is among the most abundant sources of mesenchymal stem cells (MSCs). Human adipose tissue-derived stem cells (hADSCs) are used in regenerative medicine because they are easily obtained, for example, via liposuction, and can be efficiently isolated [6]. These cells are being investigated for the treatment of various eye diseases, including glaucoma, corneal chemical burns, ocular surface reconstruction, thyroid-associated orbitopathy, age-related macular degeneration, and optic nerve damage [7]. They play a key role in growth, angiogenesis, and inflammation. The therapeutic potential of ADSCs is primarily attributed to their paracrine properties, as they secrete pro- (IL-1, IL-2, IL-6, IL-8) and anti-inflammatory (IL-4, IL-10, IL-13) interleukins, tumor necrosis factor α (TNF-α), and growth factors such as transforming growth factor α (TGF-α) and transforming growth factor β1 (TGF-β1) [8,9,10]. A study of ADSC function in a rat limbal stem cell deficiency (LSCD) model demonstrated the therapeutic potential of these cells to repair corneal damage, restore transparency, reduce inflammation, and modulate paracrine activity [11].

Another candidate is cells isolated from the human amniotic membrane, which have been used for several years to treat corneal injuries [12]. Suturing the amniotic membrane during keratoconus or refractive surgery reduces corneal opacification and keratocyte proliferation during healing, leading to improved postoperative outcomes. The amniotic membrane serves as a carrier for limbal epithelial stem cell (LESC) proliferation following reimplantation and ocular surface reconstruction in cases of limbal insufficiency. It stimulates epithelialization of the damaged cornea and conjunctiva and suppresses the inflammation, neovascularization, and scarring [12]. It also contains matrix metalloproteinases (MMPs) and tissue inhibitors of metalloproteinases (TIMP-1, -2, -3, and -4), primarily derived from epithelial cells. The amniotic membrane is a rich source of stem cells capable of proliferating and differentiating in vitro along multiple lineages. Isolation of these cells does not affect fetal or maternal tissues and therefore raises no ethical concerns.

Human amniotic epithelial cells (hAECs) and human amniotic mesenchymal stromal cells (hAMSCs) can differentiate into mesodermal, endodermal, and ectodermal lineages, enabling their broad application in regenerative medicine [13]. Among other effects, the hAEC secretome has been shown to inhibit cell migration and growth, although its mechanism of action remains unclear [14]. hAMSCs exhibit immunomodulatory properties and secrete a variety of bioactive molecules, including growth factors such as FGF2, IGF-1, HGF, and VEGF, as well as cytokines [15]. They decrease expression of the proapoptotic protein Bax and increase expression of the antiapoptotic protein Bcl-2, thereby promoting cell survival, reducing chemotherapy-induced apoptosis, stimulating tissue repair, and regulating immune responses to create an environment conducive to healing [16]. One of the most promising applications of the CM obtained from hAMSC cultures (hAMSC-CM) is in ocular tissue regeneration, for example, in the treatment of ocular surface damage caused by chemical burns. In vitro studies have shown that this CM reduces myofibroblast activation and the release of inflammatory factors, suggesting both anti-inflammatory and antifibrotic effects [17].

The efficacy of CM appears to be closely linked to the timing of its administration relative to toxic exposure. When administered simultaneously with the toxic agent, CM may provide a protective effect by inhibiting the inflammatory response and reducing early cellular damage, likely through its immunomodulatory and cytoprotective factors such as TGF-β and IL-10. However, administration of CM after toxic damage appears to support tissue repair by promoting angiogenesis (via VEGF), extracellular matrix remodeling (via MMP-2), and enhanced cell survival and migration [18]. These differences highlight the potential of CM to act both preventively and therapeutically, preventing early damage when administered immediately and promoting regeneration when applied after injury. This dual role underscores the importance of timing in CM treatment when designing therapeutic strategies for tissue injury [19].

In this study, we hypothesized that CM supports tissue regeneration through cytoprotective mechanisms. The aim of the study was to (1) evaluate differences in the efficacy of CM derived from amniotic epithelial cells (hAECs), adipose-derived mesenchymal stem cells (hADSCs), and amnion-derived cells expressing both epithelial and mesenchymal markers (hACs); and (2) compare the effects of these CM with those of BAC when co-administered or applied after prior tissue damage induced by BAC, with particular emphasis on the modulation of inflammation, apoptosis, cell cycle, proliferation, and migration of LSCs.

## 2. Materials and Methods

Limbal stem cells (LSCs) were harvested from the corneoscleral rim of a deceased donor’s eye following corneal extraction for transplantation. The study involving human cells was approved by the Bioethics Committee of the Medical University of Silesia (SUM) in Katowice (decision nos. PCN/CBN/0052/KB/26a/22 and PCN/CBN/0052/KB/26b/22). As described in our previous study [20], the isolated LSCs were cultured in vitro and identified by immunofluorescence staining for positive (p63, ABCG2) and negative (keratins CK3 and CK12) markers [20]. In the present study, the expression of the mRNAs corresponding to p63 and ABCG2 proteins, as well as to ΔNp63α isoform, was assessed by RT-qPCR. Flow cytometry was performed to evaluate the expression of surface markers characteristic of limbal mesenchymal stem cells (LMSCs)—specifically CD73, CD90, and CD105—confirming that the isolated population contained both limbal epithelial stem cells (LESCs) and LMSCs.

Human amniotic epithelial cells (hAECs) and human amniotic cell line cells (hACs) were assessed by flow cytometry for the pluripotency marker SSEA-4, epithelial markers (CK14, CK15, CK16, CK19), and mesenchymal markers (CD44, CD73, CD90, CD105). Human adipose-derived stem cells (hADSCs) were characterized by flow cytometry for mesenchymal surface markers (CD73, CD90, CD105). Conditioned media (CM) were collected from hADSC, hAEC, and hAC cultures after 24 h of incubation, when the cells had reached 70–80% confluence.

**Figure 1:**
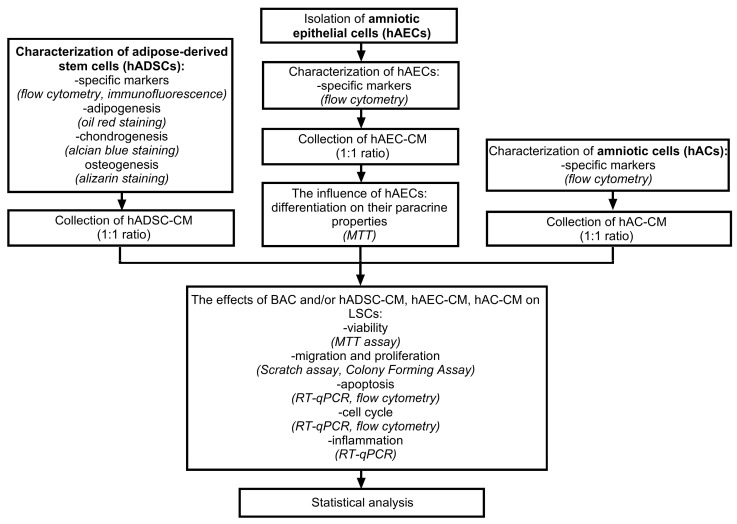
Schematic diagram of the experiment.

The viability of LSCs was assessed using the MTT assay. Cell proliferation and migration were evaluated by scratch assay and colony-forming assay. Apoptosis was examined using both RT-qPCR, to measure the expression of apoptotic genes (*BAX*, *BCL2*, *TP63*, *CASP3*, *CASP7*), and flow cytometry with Annexin V/PI staining, to identify apoptotic cells. The expression of cell cycle-related genes (*E2F2*, *CCND1*, *CCND2*, *CDKN2C*, *CDKN2A*, *CCNE1*, *CCNE2*, *RB1*) was analyzed by RT-qPCR, and the distribution of cells among different cell cycle phases was determined by flow cytometry using FxCycle staining. In addition, the effects of CM on inflammation and the expression of NLRP3 pathway-related genes (*IL-1β*, *IL-6*, *IL-4*, *IL-10*, *NLRP3*, *CASP1*, *IL-18*, *MMP3*, *MMP9*, *TIMP1*) were analyzed by RT-qPCR. A schematic representation of the experimental design is shown in Figure 1.

**Figure 2:**
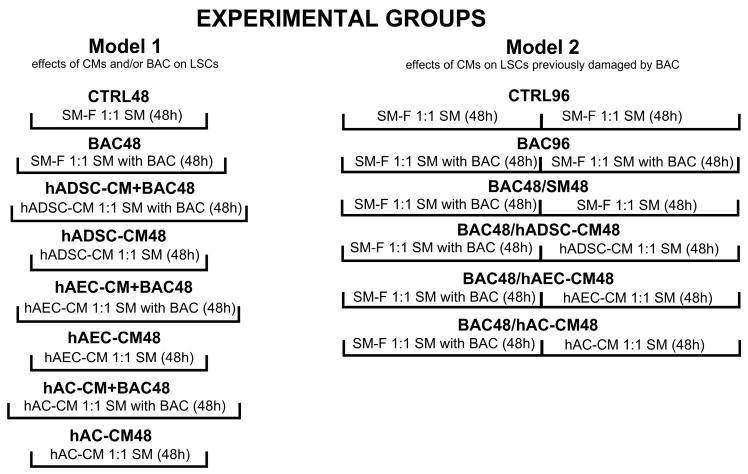
Experimental models and groups used in the study. The first model evaluated the neutralizing effect of conditioned medium (CM) on the toxic effects of concomitantly administered benzalkonium chloride (BAC). The second model involved pretreating LSCs with BAC for 48 h, followed by exposure to CM for an additional 48 h. CTRL, control group; CM, conditioned medium; SM, standard medium; SM-F, frozen standard medium.

The effects of hADSC-CM, hAEC-CM, and hAC-CM on LSCs were investigated using two experimental models (Figure 2). The first model was designed to test whether the conditioned medium could neutralize the toxic effects of benzalkonium chloride (BAC) when administered concurrently. In this model, BAC- and CM-treated cells were cultured in parallel for 48 h. The second model involved pretreating LSCs with BAC for 48 h, followed by exposure to CM for an additional 48 h, to evaluate the ability of CM to mitigate the effects of prior BAC-induced damage. The concentration of BAC used was 0.0002%. Control groups in the first and second models consisted of cells cultured in standard medium (SM): 89% DMEM/F12, 10% FBS, 1% AA, and 20 ng/mL EGF, for 48 and 96 h, respectively. In selected groups, frozen standard medium (SM-F) was used instead of CM to exclude the potential effects of storage conditions on CM properties. SM-F controls for any physicochemical alterations introduced by the freeze–thaw process, ensuring that observed cellular effects are attributable to factors specific to CM. Together, the two models provided complementary information about the cytoprotective properties of CM in the context of BAC-induced toxicity.

### 2.1. Characterization of hADSCs

hADSCs (cell line PT5006, Lonza, Basel, Switzerland) were cultured and characterized by identifying specific markers (CD73, CD90, CD105) with the use of Human Mesenchymal Stem Cell Verification Flow Kit (FMC020, R&D Systems, Minneapolis, MN, USA). The ability of hADSCs to undergo multilineage differentiation was examined using the Human Mesenchymal Stem Cell Functional Identification Kit (SC006, R&D Systems, Minneapolis, MN, USA). For this purpose, hADSCs were cultured in dedicated adipogenic, osteogenic, and chondrogenic media. The composition of each differentiation medium was consistent with the manufacturer’s protocol provided in the kit. Adipogenic differentiation was performed for 1 week, whereas osteogenic and chondrogenic differentiation continued for 2 weeks, depending on the appearance of characteristic morphological features. After this time, cells were stained by immunofluorescence for FABP4 (adipogenesis), osteocalcin (OCN; osteogenesis), and aggrecan (chondrogenesis), as well as by histochemistry with Oil Red O, Alizarin Red, and Alcian Blue.

For immunofluorescent staining, hADSCs were seeded on coverslips, fixed with 4% paraformaldehyde, and permeabilized with 0.5% Triton X-100 and Tween 20 (both from Sigma-Aldrich, St. Louis, MO, USA). After thorough washing with PBS (Corning, Corning, NY, USA), the cells were incubated with blocking buffer for 1 h, followed by additional PBS rinsing. Then, hADSCs were labeled with the primary antibody at 4 °C overnight. The samples were subsequently washed three times for 5 min each with PBS and incubated with secondary antibodies for 1 h. Cell nuclei were stained with DAPI (VECTASHIELD Vibrance Antifade Mounting Medium, Vector Laboratories, Newark, CA, USA). The stained sections were compared with the negative controls, in which the primary antibody was omitted and replaced with blocking buffer.

For histochemistry, the negative controls consisted of cells cultured in SM without differentiation factors.

Finally, all samples were observed under an Olympus IX73 microscope (Olympus, Shinjuku, Tokyo, Japan).

#### 2.1.1. Adipogenic Differentiation—Immunofluorescence Staining and Oil Red Staining

hADSCs were identified with mouse anti-fatty acid-binding protein 4 (mFABP4), a marker of adipogenic differentiation, and incubated with donkey anti-goat secondary antibody (R&D Systems, Minneapolis, MN, USA) (Supplementary Table S1). Oil Red O staining was performed by dissolving Oil Red O (Sigma-Aldrich, St. Louis, MO, USA) in distilled water to a final concentration of 0.5%. The samples were fixed in 4% formaldehyde for 20 min, washed with PBS, and stained with Oil Red O solution for 20 min at room temperature. After staining, the samples were rinsed with water to remove excess dye.

#### 2.1.2. Osteogenic Differentiation—Immunofluorescence and Alizarin Red Staining

hADSCs were stained with anti-hOsteocalcin antibody (OCN), a marker of osteogenic differentiation, and incubated with donkey anti-mouse secondary antibody (R&D Systems, Minneapolis, MN, USA) (Supplementary Table S1). For Alizarin Red staining, hADSCs were fixed in 4% paraformaldehyde, washed with PBS, and incubated in 40 mM Alizarin Red solution (Sigma-Aldrich, St. Louis, MO, USA) for 30 min at room temperature. After staining, the samples were washed with distilled water to remove excess dye.

#### 2.1.3. Chondrogenic Differentiation—Immunofluorescence Staining and Alcian Blue Staining

hADSCs were stained with anti-hAggrecan antibody, a marker of chondrogenic differentiation, and incubated with donkey anti-goat secondary antibody (R&D Systems, Minneapolis, MN, USA) (Supplementary Table S1). For Alcian Blue staining, hADSCs were cultured in a chondrogenic medium under non-adherent conditions, allowing the cells to aggregate into micropellets. After 14 days of induction, the cell pellets were fixed in 4% paraformaldehyde, embedded in paraffin, and sectioned using a microtome following standard histological procedures. To assess the production of proteoglycan-rich extracellular matrix, the sections were deparaffinized, rehydrated through a graded ethanol series, and incubated in Alcian Blue solution (Sigma-Aldrich, St. Louis, MO, USA) for 30 min at room temperature. After staining, the slides were rinsed with distilled water, dehydrated again through an ascending ethanol series, and cleared in xylene (Stanlab, Lublin, Poland) before mounting.

### 2.2. Characterization of Amniotic Cells

Two types of amniotic cells were used in the study: primary hAECs isolated from human placenta, and a cell line isolated from human amnion (T0531, ABM, Richmond, Canada). The placenta-derived hAECs exhibited a typical epithelial phenotype, whereas the hACs displayed a mixed expression profile of epithelial and mesenchymal markers, with mesenchymal characteristics predominating. This distinction was essential for assessing how specific cellular features could influence the composition and biological activity of the secretome.

#### 2.2.1. hAEC Isolation and Characterization During Culture

hAECs were isolated using a multi-step procedure that began with the collection of amniotic membrane tissue [21]. The membrane was first rinsed with PBS to remove contaminants and then subjected to enzymatic digestion with 0.05% trypsin (Gibco, Grand Island, NY, USA) to release the cells. After digestion, the cell suspension was filtered through a mesh to remove debris and subsequently cultured in SM composed of 89% Dulbecco’s Modified Eagle Medium (DMEM) with L-glutamine (Gibco, Grand Island, NY, USA), 10% fetal bovine serum (FBS, EURx, Gdańsk, Poland), 1% antibiotic-antimycotic solution (AA, Corning, USA), and 20 ng/mL epidermal growth factor (EGF; Sigma-Aldrich, St. Louis, MO, USA).

Following isolation, the cells were seeded and the culture medium was replaced. To exclude the possibility of epithelial-to-mesenchymal transition (EMT) in the examined epithelial cells, hAECs were analyzed at 72, 120, 168, and 216 h post-isolation based on their epithelial morphology and expression of specific markers (SSEA-4, CK14, CK15, CK16, CK19, CD44, CD90, CD105) using flow cytometry. This phenotypic characterization was complemented by assessing the effect of hAEC-derived secretome on LSC viability. Together, these analyses determined the culture period during which hAECs retained epithelial characteristics and the associated properties of the conditioned medium.

#### 2.2.2. Characterization of Amniotic Cell Line Cells

hACs were cultured and characterized with flow cytometry using the pluripotency marker SSEA-4, epithelial markers CK14, CK15, CK16, and CK19, and mesenchymal markers CD44, CD90, and CD105. For this purpose, hACs were enzymatically detached from the surfaces of the culture flasks, suspended in staining buffer (BD Biosciences, San Jose, CA, USA) containing the relevant fluorochrome-conjugated antibodies and, in separate test tubes, the appropriate isotype controls (Supplementary Table S1). After incubation, the cell suspension was rinsed and centrifuged (500 g, 5 min, 4 °C). Finally, the cells were suspended in labeling solution and analyzed on a flow cytometer (CytoFLEX, Beckman Coulter Brea, CA, USA).

### 2.3. Collection and Preparation of Conditioned Medium

hADSCs and amniotic-derived cells were cultured in SM containing 89% DMEM with L-glutamine (Gibco, USA), 10% FBS (EURx, Gdańsk, Poland), and 1% AA (Corning, Corning, NY, USA). hADSC-CM and hAC-CM were collected at passage 3 after several days of culture, when cells reached 70–80% confluence. The medium was replaced with fresh standard medium, and CM were collected 24 h later. Passage 3 was selected due to the stable phenotype of hADSCs and hACs, whereas hAECs underwent EMT. hAEC-CM was collected at 72, 120, 168, and 216 h post-isolation and applied to culture LSCs in the presence of BAC for 48 h in the first model, and after 48 h of BAC-induced damage in the second model. To obtain freshly secreted factors, the medium was replaced 24 h before each collection time point. The 72 h post-isolation time point was selected for hAEC-CM collection based on preliminary marker expression profiling and assessment of the phenotype-dependent effect of the hAEC secretome on the viability of LSCs exposed to BAC. Finally, CM from hADSCs, hAECs, and hACs were collected at 70–80% confluence after 24 h of incubation in fresh medium, then centrifuged at 3500 rpm for 10 min to separate cells from the supernatant. Supernatants were diluted 1:1 in culture medium containing DMEM/Nutrient Mixture F-12 (DMEM/F-12) with L-glutamine (Gibco, Grand Island, NY, USA) supplemented with 10% FBS and 1% antibiotics.

### 2.4. Identification and Culture of LSCs

LSCs were isolated by the explant method, as previously described [20]. Identification was performed using RT-qPCR to assess the expression of *TP63*, *ΔNp63α*, and *ABCG2* genes, which are characteristic of LSCs.

#### 2.4.1. Assessment of LSC Viability and Apoptosis

##### MTT Assay

LSCs were seeded in 96-well plates at a density of 10^4^ cells per well and incubated in culture medium for several days. The negative control consisted of cells treated with 0.1% Triton X-100 (Sigma-Aldrich, St. Louis, MO, USA). The MTT solution (3-[4,5-dimethylthiazol-2-yl]-2,5-diphenyltetrazolium bromide, Sigma-Aldrich, St. Louis, MO, USA) was added to the wells, and the cells were incubated for 3 h at 37 °C and 5% CO_2_ in an incubator (Sanyo MCO-19M, Osaka, Japan). After incubation, the medium was removed, and the resulting formazan crystals were dissolved by adding 100 µL of DMSO to each well. Absorbance was measured at 570 nm using a Victor Nivo plate reader (Perkin Elmer, Waltham, MA, USA). Absorbance values from the control group were assigned as 100% (CTRL = 1).

##### Annexin/PI

Apoptosis was assessed using the Annexin V/PI Apoptosis Detection Kit (BD Biosciences, San Jose, CA, USA). This method allows for the detection of early and late apoptotic cells by combining Annexin V binding and propidium iodide (PI) staining. Cells were harvested, washed with PBS, and resuspended in binding buffer. Annexin V was added and incubated for 15 min at room temperature in the dark. PI was then added to stain the DNA of late apoptotic and necrotic cells. Cells were washed in binding buffer, and samples were immediately analyzed by flow cytometry to determine the percentages of viable, early apoptotic, late apoptotic, and necrotic cells.

#### 2.4.2. Cell Proliferation—Colony-Forming Assay

The colony-forming assay (CFA) is a key method to assess the self-renewal and proliferation capacity of stem cells. This assay provides insight into how CM modulates the regenerative potential of LSCs, which is essential for restoring corneal integrity following BAC exposure. To perform the CFA, cells were seeded at a density of 500 cells per well in 6-well plates and incubated for 10 days at 37 °C in a 5% CO_2_ atmosphere. Depending on the group, CM or BAC was added to the culture every other day during the incubation period. Each condition was analyzed in triplicate. After incubation, cells were fixed with 4% formaldehyde for 25 min and stained with 0.5% crystal violet solution (Sigma-Aldrich, St. Louis, MO, USA) for 10 min to assess proliferation and morphology.

#### 2.4.3. Cell Proliferation and Migration—Scratch Assay

Scratch assay was performed to evaluate the effects of hADSC-CM, hAEC-CM, and hAC-CM on LSC migration. LSCs were seeded into 6-well plates and cultured until reaching 100% confluence. A cell-free area was created using micropipette tips. The cells were then rinsed with PBS and incubated in the appropriate medium for each group. Images were captured with an Olympus IX73 microscope (Olympus, Shinjuku, Tokyo, Japan) immediately after scratch induction (0 h) and at 6, 12, 18, and 24 h. The cell-free area was quantified using ImageJ software (v. 1.52a, USA), (n = 6).

#### 2.4.4. Cell Cycle—Flow Cytometry

LSCs were stained with FxCycle PI/RNase Staining Solution (Gibco, Grand Island, NY, USA) to analyze the cell cycle. This method enables assessment of DNA content, enabling identification of G1, S, and G2 phases. Cells were harvested and washed with PBS to remove residual culture medium. After centrifugation, the cell pellet was resuspended in FxCycle PI/RNase staining solution. Cells were incubated for 30 min at room temperature in the dark to allow PI to bind to DNA. Samples were then analyzed by flow cytometry. PI fluorescence was measured to assess the DNA content of the cells and to determine the number of cells in each phase of the cell cycle (G1, S, and G2). All groups were analyzed in triplicate (n = 3).

### 2.5. Gene Expression—RT-qPCR

LSCs were cultured in the presence of BAC and CM in both models to evaluate the effects of hADSC-, hAEC-, hAC-derived secretomes on gene expression using RT-qPCR. Prior to RT-qPCR, total RNA was extracted from LSC pellets using RNA Extracol (EURx, Gdańsk, Poland) according to the manufacturer’s instructions. RNA concentration and purity were measured using a NanoDrop 2000 spectrophotometer (Thermo Fisher Scientific, Waltham, MA, USA) (n = 6). Expression of genes related to the cell cycle (*CDKN2C*, *CDKN2A*, *CCND1*, *CCND2*, *CCNE1*, *CCNE2*, *RB1*, *E2F2*), apoptosis (*BAX*, *BCL2*, *CASP3*, *CASP7*, *TP53*), and inflammation (*IL1β*, *IL6*, *IL4*, *IL10*), including the NLRP3 pathway and MMPs (*NLRP3*, *IL1β*, *IL18*, *CASP1*, *MMP3*, *MMP9*, *TIMP1*), was analyzed using RT-qPCR with SYBR Green (GoTaq 1-Step RT-qPCR System, Promega, Madison, WI, USA) on a LightCycler 96 Instrument (Roche, Basel, Switzerland). *ACTβ* served as the reference gene. Primer sequences used in the experiments are listed in Supplementary Table S2. To visualize the RT-qPCR products, electrophoresis was performed on a 2% agarose gel.

### 2.6. Statistical Analysis

Statistical analysis was performed using Statistica version 13.3 (TIBCO Software Inc., Palo Alto, CA, USA). Study groups were compared using analysis of variance (ANOVA) followed by Tukey’s post hoc test for normally distributed data. Results were considered statistically significant at *p* < 0.05.

## 3. Results

### 3.1. LSC Identification

**Figure 3:**
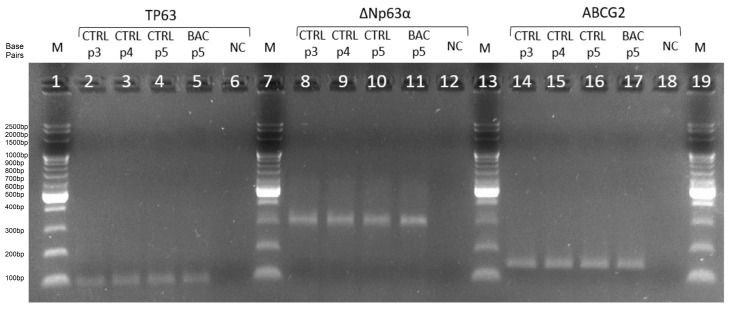
Agarose gel electropherogram of amplification products of *TP63* (96 bp), *ΔNp63α* (299 bp), and *ABCG2* (144 bp) in the third (p3), fourth (p4), and fifth (p5) passages. CTRL, control group; BAC, benzalkonium chloride; NC, negative control; M, DNA ladder; p3/p4/p5, passages 3/4/5.

We assessed the expression of *ABCG2*, *TP63*, and *ΔNp63α* genes by RT-PCR at passages 3, 4, and 5 to confirm the presence of LSCs in culture. Expression of all three markers was detected and remained at comparable levels across passages, confirming the maintenance of the LSC phenotype. RT-qPCR products were visualized by agarose gel electrophoresis (Figure 3).

### 3.2. hADSC Characteristics

**Figure 4:**
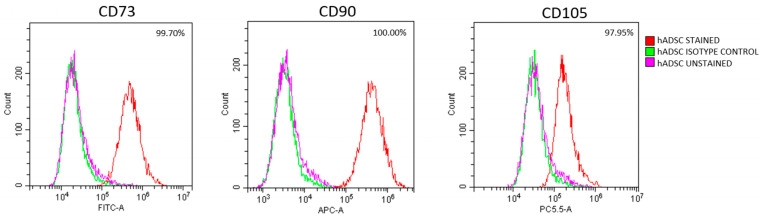
hADSC identification at passage 3. Representative flow cytometry results confirming the mesenchymal phenotype of hADSCs, based on high expression of CD73, CD90, and CD105.

At the beginning of the experiment, cells at passage 3 exhibited typical mesenchymal stem cell characteristics and strong expression of the surface markers CD73 (99.70%), CD90 (100%), and CD105 (97.95%) (Figure 4). Lipid accumulation visualized by Oil Red O staining after 7 days confirmed adipogenic differentiation, which was further supported by the immunofluorescent detection of fatty acid-binding protein 4 (FABP4), a marker specific to adipocytes. Osteogenic potential was demonstrated by the formation of mineralized areas after 14 days, confirmed by Alizarin Red staining and immunofluorescent detection of osteocalcin (OCN). Chondrogenic differentiation was evidenced by Alcian Blue staining, showing proteoglycan deposition in the extracellular matrix after 14 days. The presence of aggrecan, detected by immunofluorescence, further confirmed cartilage matrix formation (Supplementary Figure S1).

### 3.3. Characteristics of Amniotic Cells

**Figure 5:**
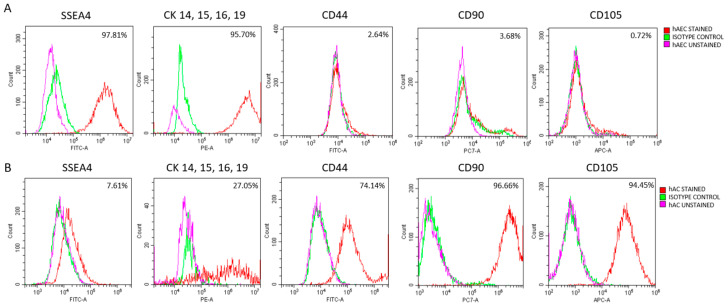
Characterization of amniotic cells by flow cytometric analysis of pluripotency (SSEA4), epithelial (CKs), and mesenchymal (CD44, CD90, CD105) markers. (**A**) hAECs analyzed 72 h after isolation. (**B**) hACs analyzed at passage 3. Percentages indicate the proportion of cells positive for each marker.

Up to 72 h after isolation, hAECs showed high expression of epithelial markers CK14, CK15, CK16, and CK19) as well as the pluripotency marker SSEA4, with no detectable expression of mesenchymal markers CD44, CD90, or CD105 (Figure 5A). During subsequent culture, a shift toward a mesenchymal-like phenotype became evident. The expression of epithelial markers, such as cytokeratins (CKs), and the pluripotency marker SSEA4 gradually decreased, whereas the expression of mesenchymal markers, including CD44, CD90, and CD105, increased (Supplementary Figure S2). These observations indicated that hAECs from early culture (up to 72 h) exhibited the most pronounced epithelial phenotype.

In preliminary studies, we observed that, in the first model, hAEC-CM collected between 72 and 216 h post-isolation did not significantly affect LSC viability when used alone (Supplementary Figure S3). LSC viability after CM treatment was comparable to that of the control group, regardless of the changes in marker expression observed after 72 h. However, the addition of BAC significantly decreased viability across all groups.

In the second model, LSCs cultured with hAEC-CM exhibited improved viability compared to the BAC96 group, although the level was comparable to that observed in the BAC48/SM48 group, reflecting spontaneous cell regeneration after BAC withdrawal. Overall, hAEC-CM collected between 72 and 216 h post-isolation did not produce significantly different effects on LSC viability, which was particularly evident in the first model, where CM and LSCs were applied simultaneously. This suggests that the phenotypic differences observed during that period were not yet sufficient to alter the biological activity of the hAEC secretome (Supplementary Figure S3). Nevertheless, the CM collected at 72 h post-isolation was selected for further experiments, since at that time point hAECs still exhibited a distinct epithelial phenotype, clearly different from the marker profile of hACs.

hACs were identified based on the expression of typical mesenchymal and epithelial markers (Figure 5B). In contrast to hAECs, hACs maintained a stable mesenchymal profile throughout the culture period (passages 2–6). Notably, distinct CK expression was observed in approximately 27% of examined hACs.

### 3.4. Impact of BAC and hADSC-CM, hAEC-CM, hAC-CM on LSC Viability

To evaluate the potential protective effects of different stem cell-derived conditioned media against BAC-induced cytotoxicity, LSCs were exposed to treatment conditions in two experimental models. Cell viability was assessed using the MTT assay after incubation for 48 h in the first model and for 96 h in the second model.

#### 3.4.1. Model 1

**Figure 6:**
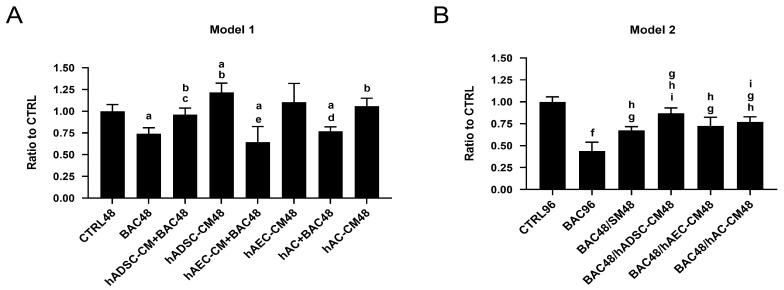
Cell viability of LSCs treated with BAC and/or CM in two different models. (**A**) After 48 h co-exposure to BAC and hADSC-CM, hAEC-CM, or hAC-CM (model 1). Statistically significant (*p* < 0.05) as compared to ^a^ CTRL48, ^b^ BAC48, ^c^ hADSC-CM48, ^d^ hAC-CM48, and ^e^ hAEC-CM48. (**B**) After subsequent (48 h/48 h) exposure to BAC and hADSC-CM, hAEC-CM, or hAC-CM (model 2). Statistically significant (*p* < 0.05) as compared to ^f^ vs. all groups, ^g^ CTRL96, ^h^ BAC96, and ^i^ BAC48/SM48.

After 48 h of treatment, BAC significantly decreased LSC viability, confirming its cytotoxic effect compared to the CTRL48 group. When LSCs were exposed simultaneously to BAC and CM, all CM (hADSC-CM, hAEC-CM, hAC-CM) improved viability compared to BAC alone (Figure 6A). Notably, hADSC-CM significantly increased LSC viability above control levels, whereas hAEC-CM and hAC-CM restored viability to levels similar to the control.

#### 3.4.2. Model 2

In the second model, 48 h BAC exposure resulted in reduced LSC viability. After BAC withdrawal, treatment with hADSC-CM, hAEC-CM, and hAC-CM improved viability compared to the BAC group. A similar recovery in the BAC48/SM48 group suggests that part of the effect was due to the intrinsic regenerative capacity of LSCs. Among the CM tested, hADSC-CM exhibited the strongest cytoprotective effect. Differences were statistically significant when comparing BAC48/hADSC-CM48 or BAC48/hAC-CM on the one hand, and BAC48/SM48 on the other, suggesting that CM contributed to enhanced recovery (Figure 6B).

### 3.5. Cell Proliferation and Migration

The dynamics of cell proliferation and migration were assessed using a scratch assay at five time points: 0, 6, 12, 18, and 24 h after cell layer disruption. The 0 h time point served as a baseline reference immediately after scratch creation, before any observable migration or proliferation could occur. The rate of wound closure was used as an indirect measure of cellular migratory capacity under different treatment conditions.

#### 3.5.1. Model 1

All groups were normalized to their respective scratch areas measured at 0 h (set as fold change = 1), which corresponded to the time of scratch creation and reflected the baseline wound size before any migration or proliferation occurred. Stable cell migration and proliferation were observed in all groups as early as 6 and 12 h after scratch induction. This effect became increasingly pronounced over time in the control and BAC48 groups. The differences were not statistically significant.

**Figure 7:**
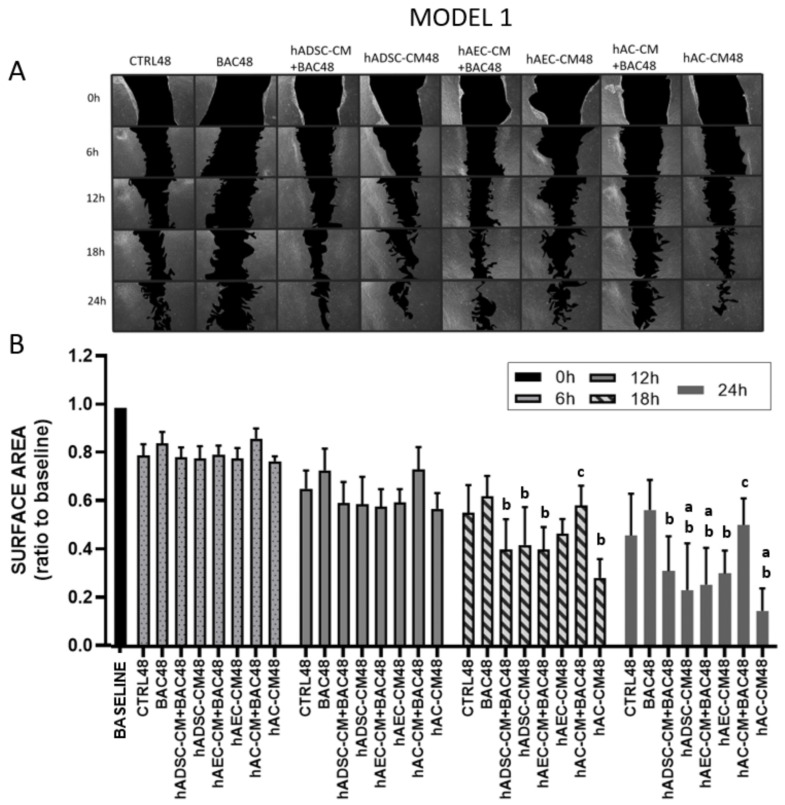
LSC proliferation and migration in the first model after 6, 12, 18, and 24 h (scratch assay). The initial scratch area at the beginning of the experiment (0 h) was described as the baseline, which was assigned a value of 1. During the test, the scratch area is inversely proportional to the cell activity (proliferation and/or migration). (**A**) Representative image of a scratched LSC monolayer, where acellular areas are black and cell monolayers are gray. (**B**) Scratch area as a ratio of the area measured at time point 0 h. Statistically significant (*p* < 0.05) as compared to ^a^ CTRL48, ^b^ BAC48, and ^c^ hAC-CM48.

From 18 h onward, a statistically significant increase in the rate of wound closure was observed in all CM- and CM+BAC-treated groups, except for hAC-CM+BAC when compared to both the BAC48 and CTRL48 groups. Notably, hAC-CM alone accelerated wound closure beyond control levels, but this effect was significantly diminished when co-administered with BAC. These trends became more pronounced at the 24 h time point (Figure 7).

#### 3.5.2. Model 2

**Figure 8:**
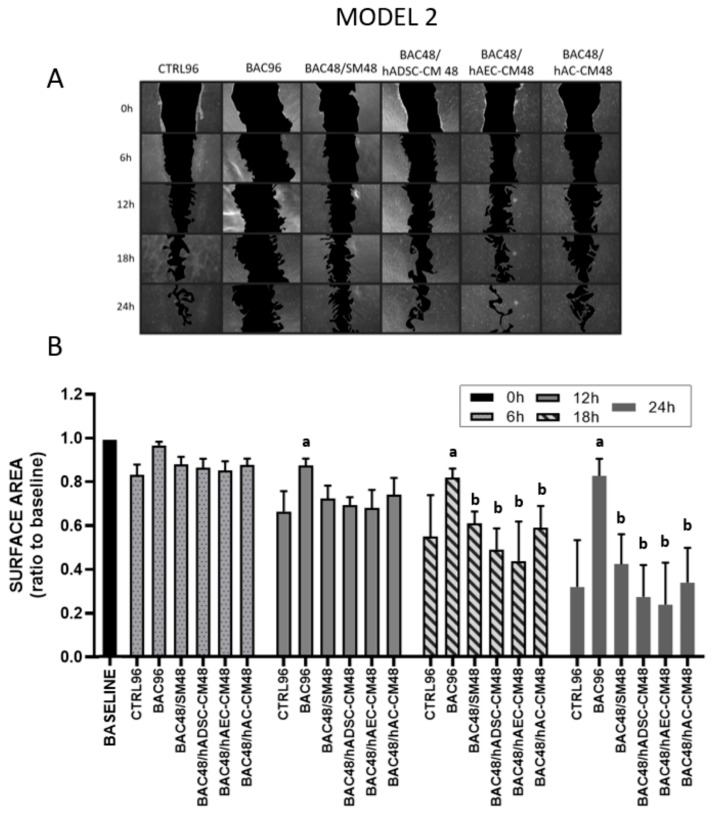
LSC proliferation and migration assay in the second model after 6, 12, 18, and 24 h. The initial scratch area at the beginning of the experiment (0 h) was described as the baseline, which was assigned a value of 1. During the test, the scratch area is inversely proportional to the cell activity (proliferation and/or migration). (**A**) Representative image of a scratched LSC monolayer, where acellular areas are black and cell monolayers are gray. (**B**) Scratch area as a ratio of the area measured at time point 0 h. Statistically significant (*p* < 0.05) as compared to ^a^ CTRL96, and ^b^ BAC96.

Stable cell migration and proliferation were observed in almost all groups at 6 and 12 h after scratch induction. At 12, 18, and 24 h onward, LSCs treated with BAC alone (BAC96) showed significantly inhibited proliferation/migration compared to the control. Intoxicated LSCs treated for 48 h with CM showed comparable recovery to those treated with standard medium (SM), indicating that the regenerative effect of CM was limited following BAC-induced damage (Figure 8).

### 3.6. Cell Proliferation and Colony Formation

#### 3.6.1. Model 1

**Figure 9:**
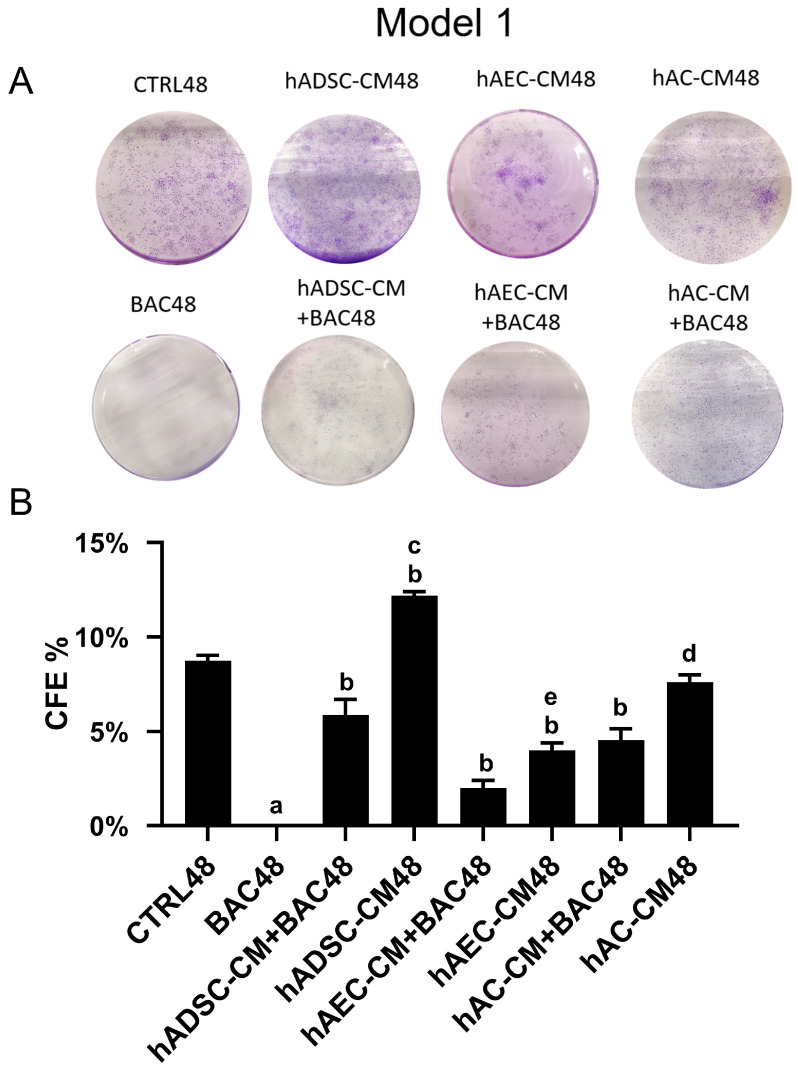
Colony-forming efficiency based on the total number of colonies in the first model. (**A**) Representative image of the colony-forming assay. (**B**) Percentage of seeded cells forming colonies (CFE% in CTRL48 = 8.73%). Statistically significant (*p* < 0.05) as compared to ^a^ all groups, ^b^ CTRL48, ^c^ hADSC-CM+BAC48, ^d^ hAC-CM+BAC48, and ^e^ hAEC-CM+BAC48.

The colony-forming assay revealed generally low clonogenic survival across all groups (Figure 9). Colony formation was completely absent in the BAC48 group. Treatment with hADSC-CM, hAEC-CM, or hAC-CM significantly increased colony formation, indicating that all CM—particularly hADSC-CM—promote cell survival. However, colony numbers decreased when CM were combined with BAC. In all groups, the percentage of colony formation did not exceed 13%.

#### 3.6.2. Model 2

**Figure 10:**
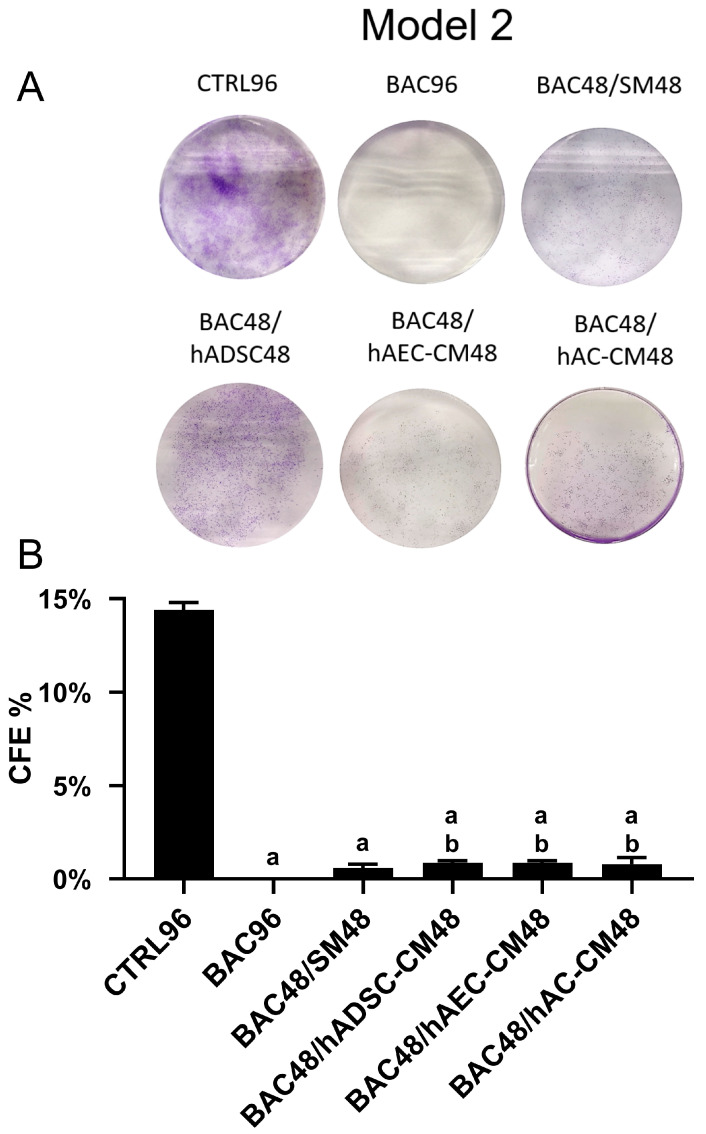
Colony-forming efficiency based on the total number of colonies in the second model. (**A**) Representative image of the colony-forming assay. (**B**) Percentage of seeded cells forming colonies (CFE% in the CTRL96 = 14.4%). Statistically significant (*p* < 0.05) as compared to ^a^ CTRL96 and ^b^ BAC96.

The complete absence of colony formation in the BAC group indicates long-lasting cellular damage caused by BAC exposure for 96 h (Figure 10). Although administration of hADSC-CM, hAC-CM, and hAEC-CM after BAC removal statistically increased the number of colonies within 48 h, it still remained significantly lower than in the control and higher than in the BAC48/SM48 group, although the difference was minimal.

### 3.7. Cell Cycle Analysis

Flow cytometric analysis of the cell cycle showed no significant differences among study groups in either the first or second experimental models (Supplementary Figure S4).

#### 3.7.1. Model 1

**Figure 11:**
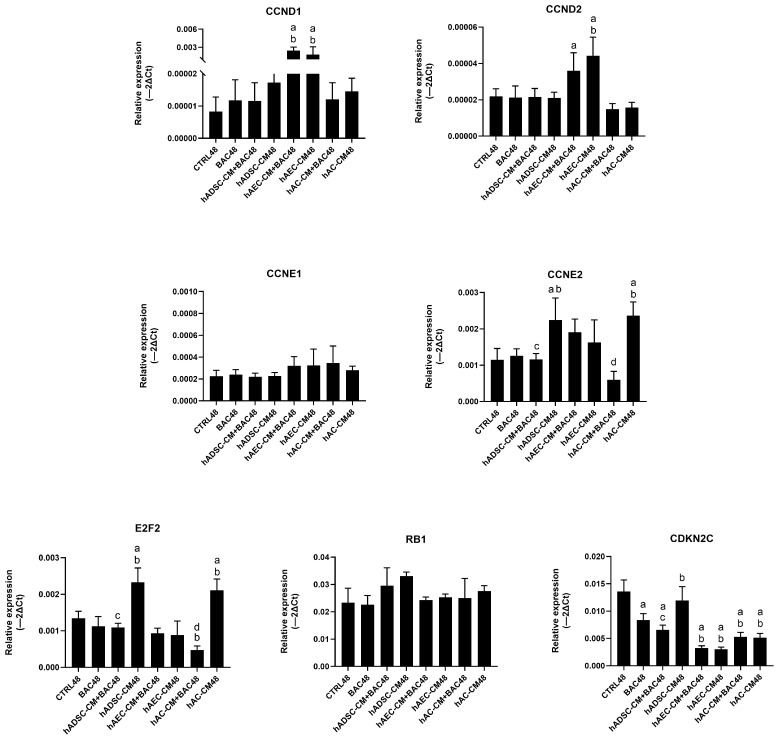
Changes in cell cycle-related gene expression in the first model presented as relative gene expression. Statistically significant (*p* < 0.05) as compared to ^a^ CTRL48, ^b^ BAC48, ^c^ hADSC-CM48, and ^d^ hAC-CM48.

Expression levels of most cell cycle-related genes remained comparable to the control group, except for CDKN2C. Levels of *E2F2* and *CCNE2* were higher in groups treated with hADSC-CM and hAC-CM than in the corresponding BAC-treated groups (hADSC-CM+BAC and hAC-CM+BAC), indicating that BAC attenuated CM-induced upregulation (Figure 11). These findings are consistent with the subtle effects observed in flow cytometry (Supplementary Figure S4).

#### 3.7.2. Model 2

**Figure 12:**
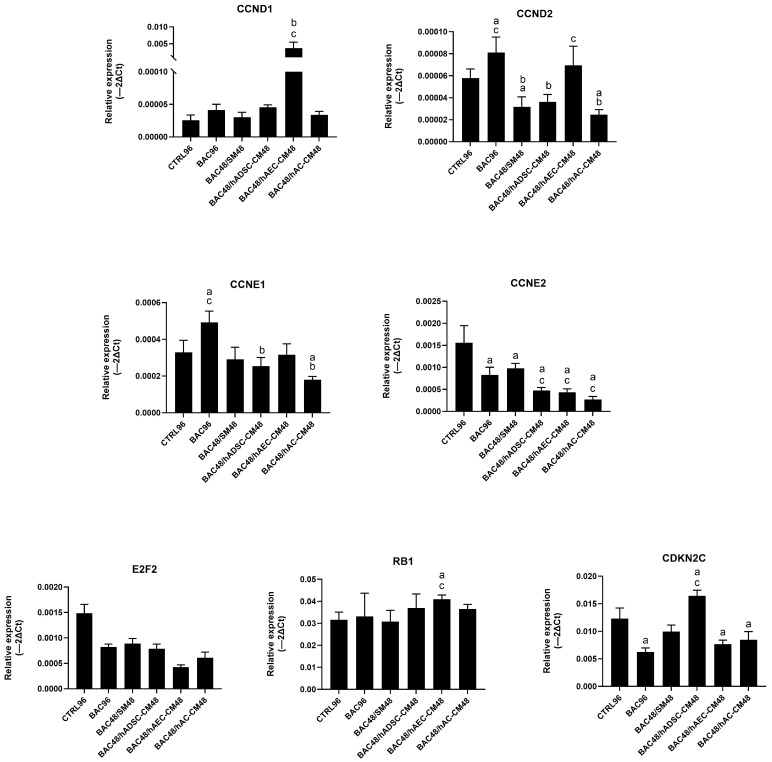
Changes in cell cycle-related gene expression in the second model are presented as relative gene expression. Statistically significant (*p* < 0.05) as compared to ^a^ CTRL96, ^b^ BAC96, and ^c^ BAC48/SM48.

Analysis revealed only modest changes in the regulation of cell cycle gene expression across groups. Treatment with CM induced modest, group-dependent changes in gene expression—particularly of *CCND2*, *CCNE1*, *CCNE2*, and *CDKN2C*—but without pronounced upregulation (Figure 12). Overall, these results suggest a limited and controlled modulation of the cell cycle.

### 3.8. Comparative Analysis of Antiapoptotic Potential of CM

#### 3.8.1. Model 1

**Figure 13:**
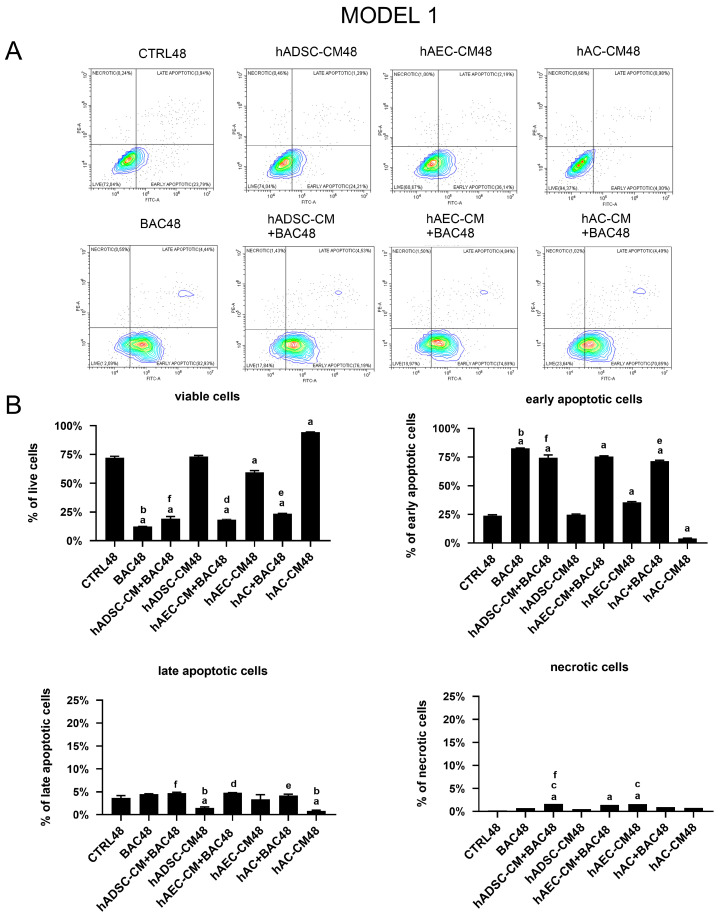
Viability and apoptosis assessment in the first model. (**A**) Representative contour plots of Annexin V/PI-stained LSCs, showing distribution of viable, early apoptotic, late apoptotic, and necrotic cells. (**B**) Percentage of cells in populations of viable, early apoptotic, late apoptotic, and necrotic cells across experimental groups. Statistically significant (*p* < 0.05) as compared to ^a^ CTRL48, ^b^ all groups, ^c^ BAC48, ^d^ hAEC-CM48, ^e^ hAC-CM48, and ^f^ hADSC-CM48.

Exposure to BAC induced a significant reduction in the number of viable cells, regardless of whether BAC was applied alone or in combination with CM. There was also an increase in the number of early apoptotic cells compared to all other groups, highlighting the pro-apoptotic effect of BAC (Figure 13).

**Figure 14:**
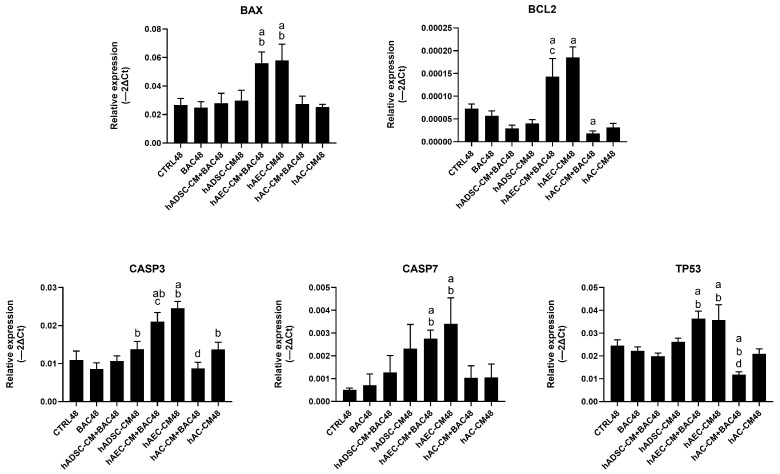
Changes in apoptosis-related gene expression in the first model are presented as relative gene expression. Statistically significant (*p* < 0.05) as compared to ^a^ CTRL48, ^b^ BAC48, ^c^ hAEC-CM48, and ^d^ hAC-CM48.

Expression of apoptosis-related genes, including *BAX*, *BCL2*, *TP53*, *CASP3*, and *CASP7*, was analyzed (Figure 14). The analysis showed a trend towards upregulation of pro-apoptotic genes, particularly *CASP7*, in cells treated with CM+BAC (vs CTRL and BAC). This effect was most notable in the hAEC-CM48 and hAEC-CM+BAC48 groups, which also showed increased expression of *BAX*, *CASP3*, *TP53*, and *BCL2*. Interestingly, the upregulation of *CASP7* and *CASP3* in the BAC+CM groups may explain the elevated levels of early apoptotic cells seen in flow cytometry, despite the overall protective effect of CM on cell viability. In contrast, the hAC-CM+BAC48 group was associated with reduced expression of *CASP3* and *TP53* (Figure 14).

#### 3.8.2. Model 2

**Figure 15:**
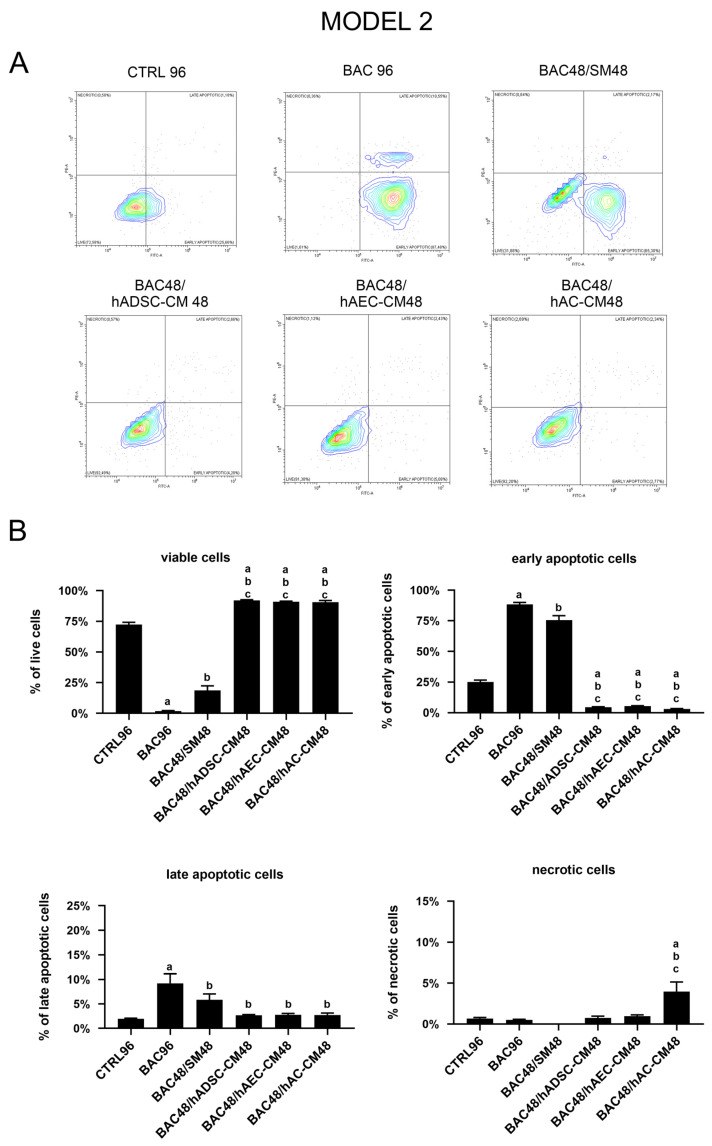
Viability and apoptosis assessment in the second model. (**A**) Representative contour plots of Annexin V/PI-stained LSCs, showing distribution of viable, early apoptotic, late apoptotic, and necrotic cells. (**B**) Percentage of cells in populations of viable, early apoptotic, late apoptotic, and necrotic cells across experimental groups. Statistically significant (*p* < 0.05) as compared to ^a^ CTRL96, ^b^ BAC96, and ^c^ BAC48/SM48.

In the second experimental model, the results demonstrated that BAC alone induced early apoptosis after 96 h of exposure compared to CTRL96. When BAC administration was followed by CM, despite the prior damage caused by BAC, LSCs exposed to hADSC-CM, hAC-CM, or hAEC-CM showed significantly improved viability compared to the CTRL96, BAC96, and BAC48/SM48 groups. Cessation of BAC alone had only a minor effect. In summary, all CM demonstrated a significant ability to counteract the apoptotic effects induced by BAC (Figure 15).

**Figure 16:**
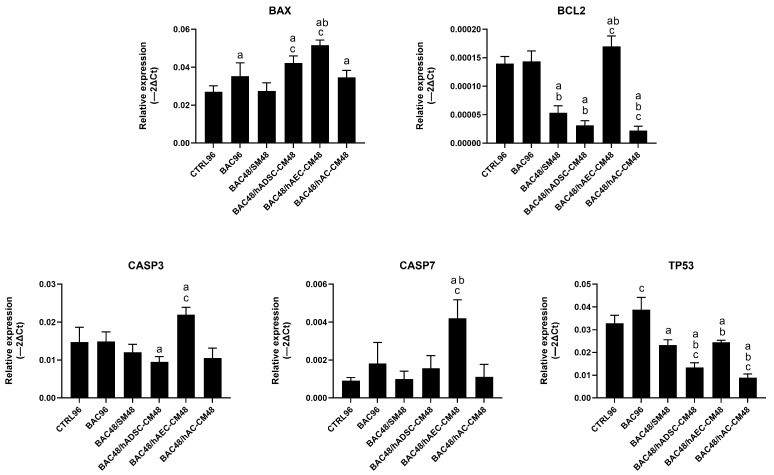
Changes in apoptosis-related gene expression in the second model are presented as relative gene expression. Statistically significant (*p* < 0.05) as compared to ^a^ CTRL96, ^b^ BAC96, and ^c^ BAC48/SM48.

Gene expression analysis did not fully reflect the antiapoptotic effects observed by flow cytometry in the second model. Treatment with hAEC-CM was associated with increased expression of several proapoptotic genes, including *BAX*, *CASP3*, and *CASP7*. In contrast, hADSC-CM and hAC-CM induced partial downregulation of *TP53*, *CASP3*, and *BCL2* (Figure 16). These discrepancies may reflect a disconnect between transcriptional activity and functional outcome, as gene expression (often low or transient) did not translate into sustained apoptosis. This suggests that CM-derived factors may inhibit downstream apoptotic processes.

### 3.9. Regulation of NLRP3 Inflammasome and Inflammatory Gene Expression

To assess the immunomodulatory effects of the tested compounds, we analyzed the expression of selected NLRP3 inflammasome-related and pro-inflammatory genes.

#### 3.9.1. Model 1

**Figure 17:**
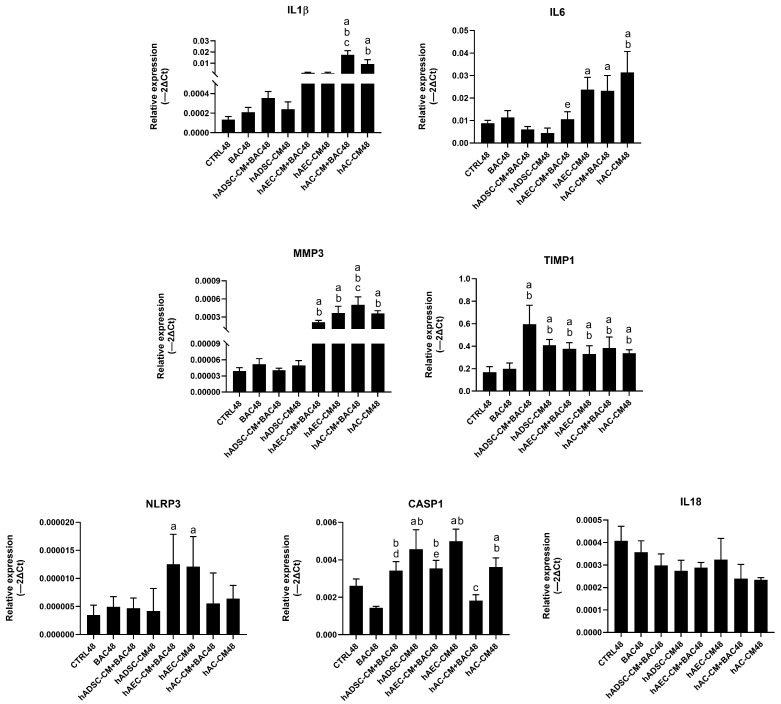
Changes in the expression of inflammatory and NLRP3 inflammasome-related genes in the first model presented as relative gene expression. Statistically significant (*p* < 0.05) as compared to ^a^ CTRL48, ^b^ BAC48, ^c^ hAC-CM48, ^d^ hADSC-CM48, ^e^ hAEC-CM48.

hAC-CM, particularly in combination with BAC, increased *IL-1β* and *IL-6* expression. In all groups, *TIMP1* was upregulated primarily by hADSC-CM, while *MMP3* expression was elevated mainly in hAEC-CM- and hAC-CM-treated cells. Overall, *TIMP1* showed markedly higher relative expression levels across groups, whereas the relative expression of other inflammatory genes remained low (Figure 17).

#### 3.9.2. Model 2

**Figure 18:**
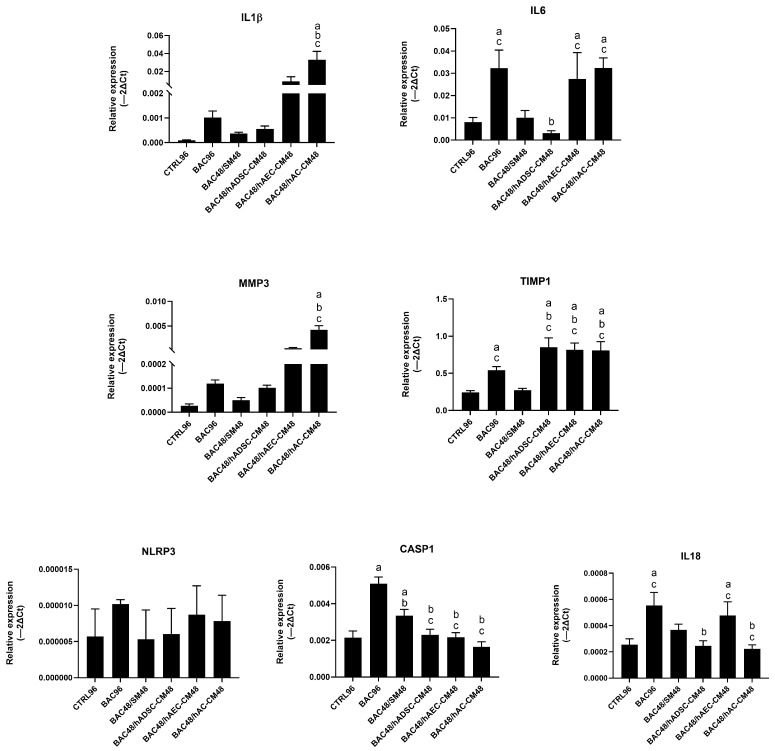
Changes in expression of inflammatory and NLRP3 inflammasome-related genes in the second model presented as relative gene expression. Statistically significant (*p* < 0.05) as compared to ^a^ CTRL96, ^b^ BAC96, and ^c^ BAC48/SM48.

In the second model, BAC (BAC96 group) induced higher expression of several inflammatory genes compared to the BAC48/SM48 group, indicating a stronger and more persistent inflammatory response when BAC exposure was prolonged. All CM upregulated *TIMP1* expression, while hAC-CM notably increased *MMP3* and interleukin levels (*IL-1β*, *IL-6*). In contrast, hADSC-CM reduced the expression of *IL-6*, *IL-18*, and *CASP1* (Figure 18).

## 4. Discussion

This study provides new insights into the effects of stem cell-derived conditioned media (CM) on limbal stem cells (LSCs) damaged by benzalkonium chloride (BAC), focusing on cell viability and inflammatory signaling. Differences in the biological activity of human adipose-derived stem cell-conditioned medium (hADSC-CM), human amniotic epithelial cell-conditioned medium (hAEC-CM), and human amniotic cell-conditioned medium (hAC-CM) reflect the cell type-specific composition and functional heterogeneity of these secretomes, highlighting their therapeutic potential in supporting LSC survival and function under stress conditions.

The hADSCs used in this study were characterized as mesenchymal stem cells based on specific surface markers and confirmed multipotency, validated by their multilineage differentiation capacity and histochemical staining typical of osteogenic, adipogenic, and chondrogenic lineages [22,23,24,25].

The hACs examined in this study exhibited typical mesenchymal markers but also partially expressed epithelial markers, including cytokeratins CK14, CK15, CK16, and CK19. This pattern indicates that the hAC population maintained an intermediate phenotype, characterized predominantly by mesenchymal but partially epithelial features. Such phenotype distinguishes hACs from typical mesenchymal cells (e.g., hADSCs) and suggests a degree of plasticity associated with their origin from the amniotic membrane [26]. In contrast, hAECs isolated directly from the amniotic membrane exhibited a distinct epithelial phenotype, characterized by the expression of cytokeratins CK14, CK15, CK16, and CK19, as well as the pluripotency marker SSEA4. These cells lacked mesenchymal marker expression, clearly distinguishing them from both hACs and hADSCs. To obtain a relatively homogeneous epithelial population and minimize the effects of epithelial–mesenchymal transition (EMT), we analyzed the temporal dynamics of epithelial and mesenchymal marker expression in hAECs at several time points (72, 120, 168, and 216 h after isolation) during in vitro culture. Specifically, we examined cytokeratins (CK) and the mesenchymal markers CD44, CD90, and CD105. The results revealed a time-dependent shift in hAEC marker expression consistent with EMT, aligning with previous reports of spontaneous EMT driven by TGF-β signaling [27]. However, the impact of these phenotypic changes on the composition and function of hAEC-CM remains poorly understood.

In our experiment, CM were collected after 24 h of culture to balance optimal accumulation of bioactive factors with cell viability. Ma et al. (2021) [28] demonstrated that CM collected from placental cells at 24 h produced similar proangiogenic and migratory effects as CM collected at 48 h in most assays. Importantly, 24 h CM from early and mid-stage placental cells (primary cytotrophoblasts and primary placenta-derived mesenchymal stem cells) often performed better. Collecting CM at 24 h also reduces issues related to nutrient depletion or waste accumulation during longer cultures, while maintaining effective levels of secreted factors [28].

Previously, we confirmed the pronounced cytotoxicity of BAC on LSCs [20]. Exposure to 0.0002% BAC caused marked reductions in cell viability, proliferation, and clonogenicity, accompanied by upregulation of genes associated with apoptosis. Notably, BAC significantly compromised LSC viability, underscoring the need for protective strategies against BAC-induced damage. To address this, we assessed the effects of hADSC-CM, hAEC-CM, and hAC-CM on BAC-treated LSCs. We selected the cytotoxic dose of BAC based on results obtained in our previous study [20]. Consistent with previous studies, the colony-forming efficiency (CFE) assay showed a complete loss of clonogenic potential following BAC exposure. Treatment with CM led to a partial restoration of this potential, measured by CFE values of the order of several percent. The approximately 10% CFE observed in the control group aligns with the threshold previously described as minimally sufficient to preserve LSC regenerative capacity [20].

In the first experimental model, where hADSC-CM, hAEC-CM, and hAC-CM were administered simultaneously with BAC, all tested secretomes demonstrated protective and regenerative effects on LSCs, as evidenced by improved viability, proliferation, and migration compared with BAC treatment. However, hADSC-CM consistently proved to be the most effective, showing the strongest enhancement of metabolic activity, scratch closure, and colony-forming efficiency. hADSC-CM significantly increased LSC viability, demonstrating a robust cytoprotective effect. This finding was further supported by assays showing that hADSC-CM accelerated wound closure and restored clonogenic potential, even in the presence of BAC. These findings align with previous studies showing that hADSC-derived factors, including extracellular vesicles and growth factors such as IGF-1 and TGF-β, stimulate corneal epithelial proliferation and maintain stemness [29,30,31]. hAEC-CM exhibited a more stabilizing effect, maintaining viability at control levels while moderately promoting wound closure and colony formation. Its effect appeared to be more protective than proliferative, consistent with reports suggesting that hAEC-CM promotes tissue repair via anti-inflammatory and survival pathways, such as ERK, JNK, and AKT signaling [32,33]. Although its regenerative potential was lower than that of hADSC-CM, hAEC-CM effectively countered BAC-induced cytotoxicity, highlighting its therapeutic potential. hAC-CM exhibited regenerative effects comparable to those of hADSC-CM and hAEC-CM, promoting LSC survival, migration, and partial restoration of clonogenic potential after BAC exposure. Previous studies indicate that hAC-CM effects may vary depending on cell type and injury context, potentially mediated by LOXL2 and other matrix remodeling enzymes [34,35].

In the second experimental model, aimed at assessing the regenerative potential of CM after BAC-induced damage, CM treatment produced generally moderate effects, resembling the natural regenerative capacity of LSCs after BAC withdrawal. This indicates that part of the observed recovery likely reflects the inherent regenerative capacity of LSCs, rather than a specific regenerative effect of CM [36,37]. Nevertheless, hADSC-CM and hAC-CM enhanced cell viability after BAC withdrawal.

LSCs treated with CM exhibited enhanced migration relative to BAC-exposed LSCs, although this improvement was similar to that observed in LSCs cultured in standard medium after BAC exposure. These results indicate that LSCs regain migratory function following removal of cytotoxic stress; however, the additional benefit of CM in this regenerative context appears limited. In other studies on corneal repair using MSC-CM, the timing of treatment and the severity of damage significantly influenced outcomes [38,39]. CFE assays revealed that, although CM treatment improved clonogenic potential relative to the BAC group, the number of colonies remained substantially reduced. This indicates that BAC induced lasting damage to LSCs, which CM could only partially reverse. The capacity of CM to fully restore LSC function is limited in cases of severe or prolonged damage [36]. Analysis of gene expression provided additional insights. Changes were observed in specific groups (e.g., *E2F2*, *CCNE2*), but no consistent upregulation was detected. These findings suggest that, although CM may support certain aspects of cell proliferation, it does not strongly affect cell cycle regulation. Previous studies have demonstrated CM-induced epithelial proliferation mediated through the PI3K/AKT and ERK pathways [15].

CM-induced changes in cell cycle regulation were subtle and inconsistent. No significant alterations in cell cycle phase distribution were observed, and transcriptional analysis revealed only mild, non-directional modulation of cyclins and CDK inhibitors. These findings suggest that CM may affect LSC proliferation indirectly via paracrine signaling, without inducing synchronized changes in the cell cycle. This interpretation aligns with prior studies showing that MSC-derived CM can modulate proliferative signaling without markedly disrupting key regulatory points in the cell cycle [33,36,40,41]. Di Iorio et al. (2006) demonstrated that cyclin D1 expression is linked to proliferative activity in LSCs and that cyclin-dependent kinase inhibitors promote quiescence and differentiation [42]. Similarly, Espana et al. (2012) highlighted the role of cyclin E and retinoblastoma protein in the G1/S transition in maintaining stem cell homeostasis [43].

Our data indicate a selective antiapoptotic effect of the tested CM, particularly mesenchymal hADSC-CM and hAC-CM, which reduced the proportion of apoptotic cells and modulated apoptosis-related gene expression toward a pro-survival profile. Lee et al. (2021) demonstrated that ADSC-CM reduces apoptosis through the miR-221/222–PUMA/ETS-1 axis in ischemic models [36]. The antiapoptotic effect likely contributes to the improved cell viability observed in the hADSC-CM and hAC-CM groups, emphasizing their cytoprotective potential under stress conditions relevant to ocular surface injury. Interestingly, although CM reduced overall apoptosis, flow cytometry revealed an increased proportion of early apoptotic cells in CM+BAC groups. The transcriptomic upregulation of *CASP7* and *CASP3*, particularly in the hAEC-CM+BAC group, suggests that early apoptotic signaling may still be activated. This suggests that CM may delay apoptotic progression rather than fully prevent it [44]. Apoptotic modulation by hAEC-CM appears to depend on the type of target cells. This observation is consistent with previous studies reporting proapoptotic activity of hAEC-CM in cancer cells [45], contrasting with its protective role in oligodendrocytes and keratinocytes [46].

Interestingly, the transcriptional response related to apoptosis in the second model did not fully align with the flow cytometric data. Although flow cytometry indicated reduced apoptosis in CM-treated groups, hAEC-CM was associated with increased expression of proapoptotic genes (e.g., *BAX*, *CASP3*, *CASP7*), whereas hADSC-CM and hAC-CM induced partial downregulation of *TP53* and *CASP3*. These discrepancies may reflect post-transcriptional regulation or delayed gene activation. As noted by Korfali et al. [44], *CASP7* participates in the early phase of apoptosis and may be transiently activated without full execution of the death pathway. Additionally, CM may act downstream of gene transcription by modulating caspase activity or altering other signaling cascades [36,39].

Among the tested secretomes, hADSC-CM exhibited the most pronounced anti-inflammatory profile. These findings are consistent with previous reports indicating that ADSCs attenuate inflammasome signaling in inflammatory skin lesions and experimental colitis, partly through prostaglandin E2-mediated modulation of macrophage activation [38,47]. In the context of LSCs, such anti-inflammatory signaling may reduce tissue-damaging immune responses and support regenerative processes under stress conditions. hAEC-CM and hAC-CM exhibited more selective and moderate immunomodulatory activity. These effects likely result from known components of hAEC-CM, including the IL-1 receptor antagonist and IL-10, which have been shown to modulate immune responses in vitro and in vivo [32,47]. Although the effects of hAEC-CM and hAC-CM were weaker than those of hADSC-CM, this targeted activity may help subtly control immune responses. Despite modest suppression of inflammasome-related transcripts, this activity concurrently increased expression of certain pro-inflammatory cytokines, particularly *IL-1β* and *IL-6*. This dual effect may reflect the context-dependent nature of MSC-derived secretomes. This interpretation is supported by previous work showing that hAC-CM can inhibit NLRP3 inflammasome activation in monocytes via modulation of CD14/TLR4 signaling [37].

In the second model, the inflammatory response was also more pronounced, likely due to prolonged BAC exposure. hADSC-CM again exhibited the most consistent anti-inflammatory effects, reducing the expression of *IL-6*, *IL-18*, and *CASP1*. All CM upregulated TIMP1, a matrix regulator associated with tissue remodeling and inflammation resolution, suggesting their potential role in promoting repair and modulating inflammatory processes in damaged tissues [47]. In contrast, hAC-CM increased *MMP3* and pro-inflammatory cytokines *IL-1β* and *IL-6*, indicating a more dual or context-dependent role, consistent with its previously reported effects in systemic inflammation models [39]. A limitation of this study is that protein-level expression of relevant targets was not assessed.

Taken together, these findings indicate that CM promote partial functional and molecular recovery after BAC-induced damage. Their efficacy appears limited during the regenerative phase, particularly when treatment is administered after the removal of the toxic agent. Their paracrine effects—including modulation of apoptosis, inflammation, and limited proliferation—appear to exert the greatest impact under stress conditions [39,41]. Our findings demonstrate that hADSC-CM exhibits the most consistent cytoprotective and anti-inflammatory effects on LSCs, suggesting its strong therapeutic potential in regenerative ophthalmic therapies. hAEC-CM and hAC-CM induced changes comparable to those observed with hADSC-CM, although these effects were less pronounced but remained biologically consistent. These results emphasize the importance of selecting appropriate cell sources for targeted stem cell-based therapies.

Further research should focus on a detailed analysis of secretome composition, which may provide deeper insights into the mechanisms underlying intercellular communication, immune responses, and chronic inflammation. While previous studies have primarily examined the functional roles of secreted molecules, a more comprehensive approach can reveal a broader spectrum of secretome components, including exosomes, cytokines, and microRNAs, which play key roles in modulating cellular responses and signaling pathways. Examining how these molecules interact under different physiological and pathological conditions may yield new insights into disease mechanisms and help identify potential therapeutic targets [48,49].

## 5. Conclusions

Exposure to BAC induces substantial cytotoxic damage to LSCs. All tested conditioned media (hADSC-CM, hAEC-CM, hAC-CM) protect LSCs and support their regeneration. Concurrent administration of CM with BAC exposure provides greater protection than treatment applied after toxic damage. Among the tested secretomes, hADSC-CM derived from mesenchymal cells demonstrates the greatest clinical potential as a cytoprotective and immunomodulatory agent.

## Data Availability

Data is provided within the manuscript.

## Supplementary Materials

The following supporting information can be downloaded at: https://www.mdpi.com/article/10.3390/cells14221790/s1, Supplementary Figure S1: Visualization of adipogenic, osteogenic, and chondrogenic differentiation of hADSCs. Lipid accumulation (adipogenesis) was confirmed by Oil Red O staining and FABP4 expression; calcium deposits and osteocalcin (OCN) indicated osteogenic differentiation; proteoglycan-rich matrix with aggrecan expression confirmed chondrogenic lineage. Scale bars: 15 µm (FABP4, OCN, aggrecan, Oil Red O); 30 µm (Alizarin Red, Alcian Blue). Supplementary Figure S2: Time-dependent expression of specific markers CD44, CD90, CD105, SSEA4, CK14, CK15, CK16, and CK19 in hAECs analyzed by flow cytometry at 72, 120, 168, and 216 h post-isolation. Left: representative histograms showing marker expression profiles at each time point. Right: percentages of marker-positive cells over time presented as means ± SD. hAECs showed high expression of epithelial markers (cytokeratins, CK), the pluripotency marker SSEA-4, and no detectable expression of mesenchymal markers (CD44, CD90, CD105) at 72 h. A gradual shift toward a mesenchymal phenotype was observed over the 120 h culture period, consistent with EMT progression; n = 3. Supplementary Figure S3: Viability of LSCs cultured with BAC and/or hAEC-CM, depending on the exposure time. (A) hAEC-CM were collected at 72, 120, 168, or 216 h post-isolation (p-i), and then LSCs were exposed to BAC and hAEC-CM simultaneously for 48 h. Statistically significant (*p* < 0.05) as compared to: ^a^ all groups; ^b^ CTRL and hAEC-CM 72, 120, 168, and 216 h post-isolation (p-i); and ^c^ hAEC-CM 168 h p-i. (B) LSCs were first exposed to BAC for 48 h, and then incubated for 48 h with hAEC-CM collected at the indicated time points. Statistically significant (*p* < 0.05) as compared to: ^d^ BAC 96; ^e^ CTRL 96; ^f^ hAEC-CM 168 h p-i; ^g^ hAEC-CM 72 ^h^ p-i. Supplementary Figure S4: Cell cycle distribution of LSCs in models 1 and 2 assessed by flow cytometry. Bar graphs show the percentage of cells in each phase. No significant differences in cell cycle phase distribution were observed between experimental groups. Table S1: Specific antibodies used for flow cytometry and immunofluorescence. Table S2: Specific primers used in RT-qPCR.
