# Supplementary material for: Potential Therapeutic Effects of Epithelial and Mesenchymal Stem Cell Secretome in Benzalkonium Chloride-Induced Limbal Stem Cell Dysfunction
Source: Cells. 2025 Nov 14;14(22):1790. doi: 10.3390/cells14221790 (PMC12651294; doi:10.3390/cells14221790)
Supplement: Supplementary file 1 [file cells-14-01790-s001.zip › cells-3919508-supplementary.pdf]

Supplementary Tables and Figures

Supplementary Table S1. Specific antibodies used for flow cytometry and immunofluorescence.

**Primary antibodies**			
Antibody name	Catalog Number	Manufacturer	Cells that were identified
FITC Mouse Anti-Human CD44	555478	BD Biosciences	hACs and hAECs
PE-Cy™7 Mouse Anti-Human CD90	561558		
APC Mouse Anti-Human CD105	562408		
PE Mouse Anti-Human Cytokeratin 14, 15, 16 and 19 Set	550953 (51-38255X)		
FITC Mouse anti-SSEA-4	560126		
CD90-APC Mouse IgG2A	kit no. FMC020	R&D	hADSCs
CD73-CFS Mouse IgG2B			
CD105-PerCP Mouse IgG1			
anti-hOsteocalcin antibody	kit no. SC006		
anti-mFABP4 antibody			
anti-hAggrecan antibody			
**Isotype controls**			
Antibody name	Catalog Number	Manufacturer	Cells that were identified
FITC Mouse IgG2b κ Isotype Control	555742	BD Biosciences	hACs and hAECs
PE-Cy™7 Mouse IgG1 κ Isotype Control	557872		
APC Mouse IgG1, κ Isotype Control	555751		
PE Mouse Anti-Human Cytokeratin 14, 15, 16 and 19 Set	550953 (51-36405X6)		
FITC Mouse IgG2b κ Isotype Control	555742		
Mouse IgG2A-APC Isotype Control	kit no. FMC020	R&D	hADSCs
Mouse IgG2B-CFS Isotype Control			
Mouse IgG1-PerCP Isotype Control			
**Secondary antibodies**			
Antibody name	Catalog Number	Manufacturer	Cells that were identified
NL557-conjugated donkey anti-goat	NL001	R&D	hADSCs
NL557-conjugated donkey anti-mouse	NL007		

Supplementary Table S2. Specific primers used in RT-qPCR.

Target gene	Description	Primer	Sequence (5'–3')	Product size [bp]	Tm [°C]	GenBank accession numbers
ACTβ	Reference gene	Forward	GACGACATGGAGAAAATCTG	131	85.5	NM_001101.5
		Reverse	ATGATCTGGGTCATCTTCTC			
ABCG2	LSC phenotyping	Forward	AAAGCCACAGAGATCATAGAG	144	75.5	OM985598.1
		Reverse	GATCTTCTTCTTCTTCTCACC			
ΔNp63α		Forward	GAGGTTGGGCTGTTCATCAT	299	82.5	NM_001329146.2
		Reverse	GTGGGAAAGAGATGGTCTGG			
TP63		Forward	CAGCCTATATGTTCAGTTCAG	96	76	NM_001329964.2
		Reverse	CAGTCCATGCTAATCTCAATC			
KRT3		Forward	ATTTGTGACTCTGAAGAAGG	101	81.5	NM_057088.3
		Reverse	TCCTTAAGAAGTCGATCTCATC			
KRT12		Forward	TCTAAAGACCCAACCAAAAC	190	81.5	NM_000223.4
		Reverse	CAGCATGTTACTCTGAAAGG			
CCND2	Cell cycle evaluation	Forward	ACTTCATTGAGCACATCTTG	117	86	NM_001759.4
		Reverse	ACATGGCAAACTTAAAGTCG			
E2F2		Forward	CAACTTTAAGGAGCAGACAG	93	85.3	XM_054334810.1
		Reverse	TATATCTGCAGGTTGTCCTC			
CDKN2C		Forward	CTGCAGGTTATGAAACTTGG	145	83.5	NM_078626.3
		Reverse	GCAAAGTCTGTAAAGTGTCC			
CCND1		Forward	GTGCCACAGATGTGAAG	192	80.5	NM_053056.3
		Reverse	CTTCGATCTGCTCCTGG			
CCNE1		Forward	AGACATACTTAAGGGATCAGC	94	81.5	XM_054322497.1
		Reverse	CACACCTCCATTAACCAATC			
CCNE2		Forward	GAAGTATACACACTTCATAGGG	146	77.5	XM_017013958.2
		Reverse	ATTTCCTCAAGTTTGGAAGC			
CDKN2A		Forward	AGCATGGAGCCTTCG	137	79.0	NM_000077.5
		Reverse	ATCATGACCTGGATCGG			
RB1		Forward	ACCAGATCATGTCAGAGAG	75	79.0	NM_001407166.1
		Reverse	TAACCTCCCAATACTCCATC			
BAX	Apoptosis evaluation	Forward	AACTGGACAGTAACATGGAG	151	86.5	NM_138764.5
		Reverse	TTGCTGGCAAAGTAGAAAAG			
BCL2		Forward	GATTGTGGCCTTCTTTGAG	164	87.1	NM_000633.3
		Reverse	GTTCCACAAAGGCATCC			
TP53		Forward	ACCTATGGAAACTACTTCCTG	99	82	NM_001276761.3
		Reverse	ACCATTGTTCAATATCGTCC			
CASP3		Forward	AAAGCACTGGAATGACATC	133	80.5	NM_001354782.2
		Reverse	CGCATCAATTCCACAATTTC			
CASP7		Forward	AAGCCATGGAGAAGAAAATG	128	80.1	NM_001267057.1
		Reverse	CCTGAATGAAGAAGAGTTTGG			
Il1β	Inflammation	Forward	CTAAACAGATGAAGTGCTCC	183	84.5	NM_000576.3
		Reverse	GGTCATTCTCCTGGAAGG			
IL6		Forward	GCAGAAAAAGGCAAAGAATC	178	83.5	NM_001371096.1
		Reverse	CTACATTTGCCGAAGAGC			
IL4		Forward	TCACATTGTCACTGCAAATC	182	82.3	NM_172348.3
		Reverse	CCTTCTCAGTTGTGTTCTTC			
IL10		Forward	GCCTTTAATAAGCTCCAAGAG	95	79.5	NM_000572.3
		Reverse	ATCTTCATTGTCATGTAGGC			
NLRP3		Forward	AGGTGTTGGAATTAGACAAC	198	88.5	XM_054334082.1
		Reverse	AATACATTTCAGACAACCCC			
CASP1		Forward	CAACTACAGAAGAGTTTGAGG	92	77.5	NM_001257118.3
		Reverse	AACATTATCTGGTGTGGAAG			
IL18		Forward	CCTTTAAGGAAATGAATCCTCC	95	79.5	NM_001243211.2
		Reverse	CATCTTATTATCATGTCCTGGG			
MMP3		Forward	GCAGTTAGAGAACATGGAG	155	83	NM_002422.5
		Reverse	ACGAGAAATAAATTGGTCCC			
MMP9		Forward	AAGGATGGGAAGTACTGG	151	75.5	NM_004994.3
		Reverse	GCCCAGAGAAGAAGAAAAG			
TIMP1		Forward	CACCTTATACCAGCGTTATG	168	87	NM_003254.3
		Reverse	TTTCCAGCAATGAGAAACTC			

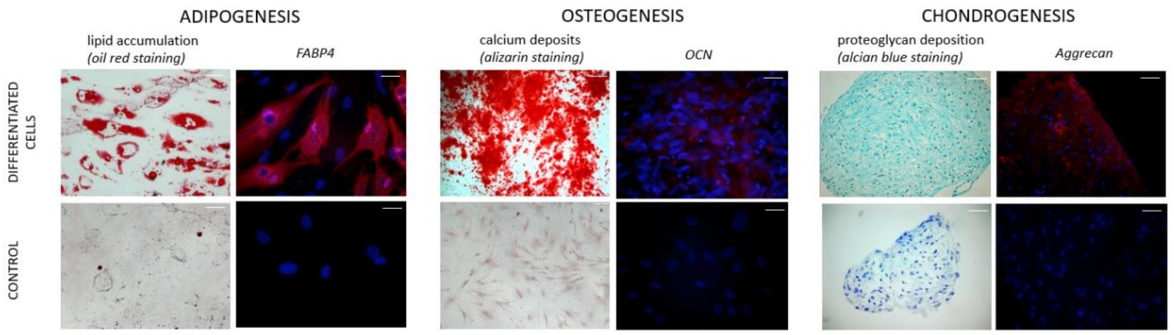

Supplementary Figure S1. Visualization of adipogenic, osteogenic, and chondrogenic differentiation of hADSCs. Lipid accumulation (adipogenesis) was confirmed by Oil Red O staining and FABP4 expression; calcium deposits and osteocalcin (OCN) indicated osteogenic differentiation; proteoglycan-rich matrix with aggrecan expression confirmed chondrogenic lineage. Scale bars: 15 µm (FABP4, OCN, aggrecan, Oil Red O); 30 µm (Alizarin Red, Alcian Blue).

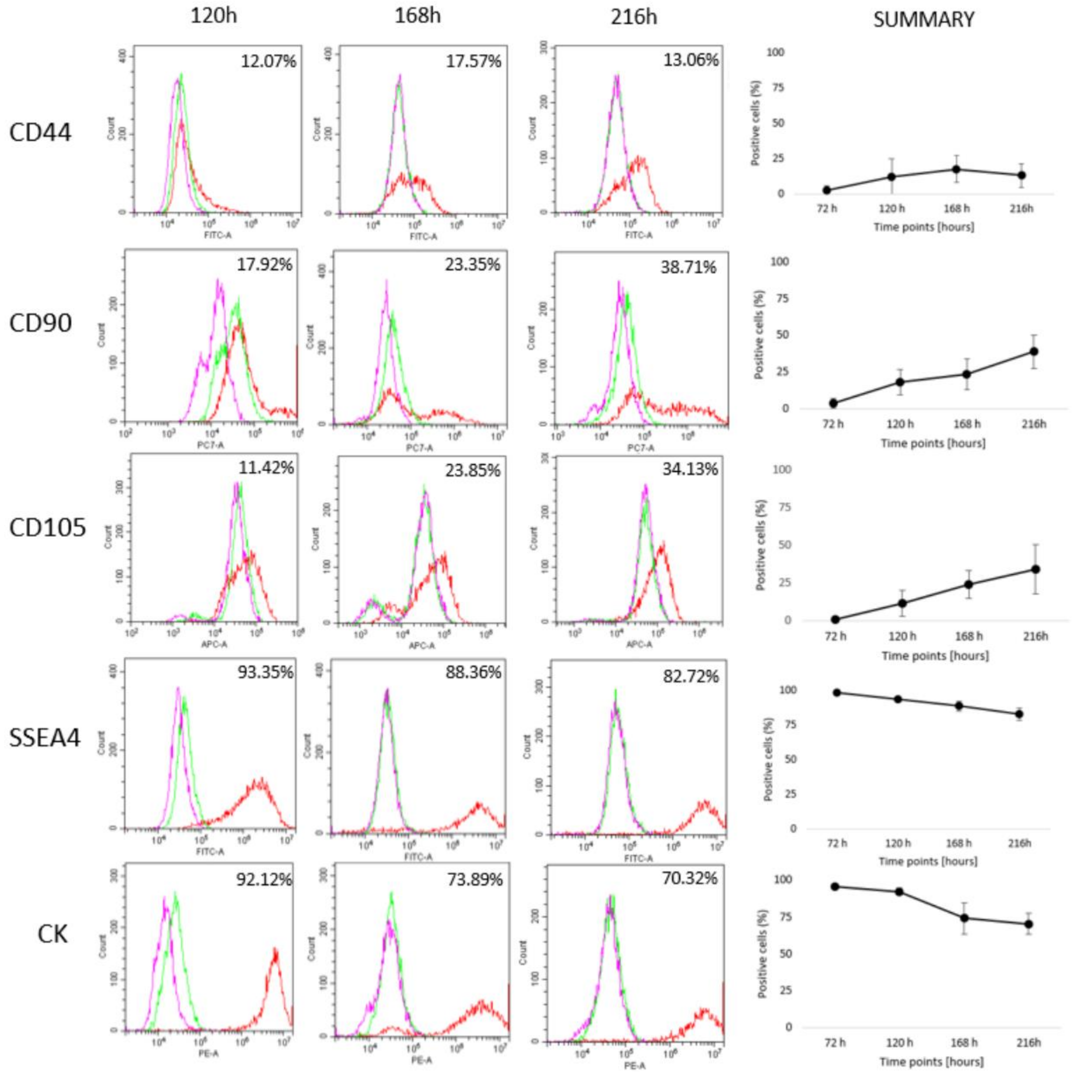

Supplementary Figure S2. Time-dependent expression of specific markers CD44, CD90, CD105, SSEA4, CK14, CK15, CK16, and CK19 in hAECs analyzed by flow cytometry at 72, 120, 168, and 216 h post-isolation. Left: representative histograms showing marker expression profiles at each time point. Right: percentages of marker-positive cells over time presented as means ± SD. hAECs showed high expression of epithelial markers (cytokeratins, CK), the pluripotency marker SSEA-4, and no detectable expression of mesenchymal markers (CD44, CD90, CD105) at 72 h. A gradual shift toward a mesenchymal phenotype was observed over the 120 h culture period, consistent with EMT progression; n = 3.

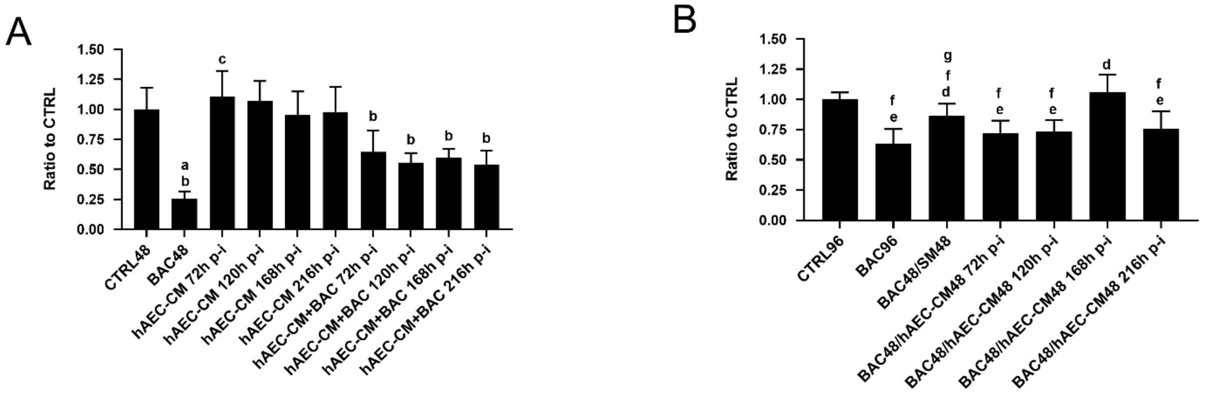

Supplementary Figure S3. Viability of LSCs cultured with BAC and/or hAEC-CM, depending on the exposure time. (**A**) hAEC-CM was collected at 72, 120, 168, or 216 h post-isolation (p-i), and then LSCs were exposed to BAC and hAEC-CM simultaneously for 48 h. Statistically significant ($p < 0.05$) as compared to: [a] all groups; [b] CTRL and hAEC-CM 72, 120, 168, and 216 h post-isolation (p-i); and [c] hAEC-CM 168 h p-i. (**B**) LSCs were first exposed to BAC for 48 h, a then incubated for 48 h with hAEC-CM collected at the indicated time points. Statistically significant ($p < 0.05$) as compared to: [d] BAC 96; [e] CTRL 96; [f] hAEC-CM 168 h p-i; [g] hAEC-CM 72 h p-i.

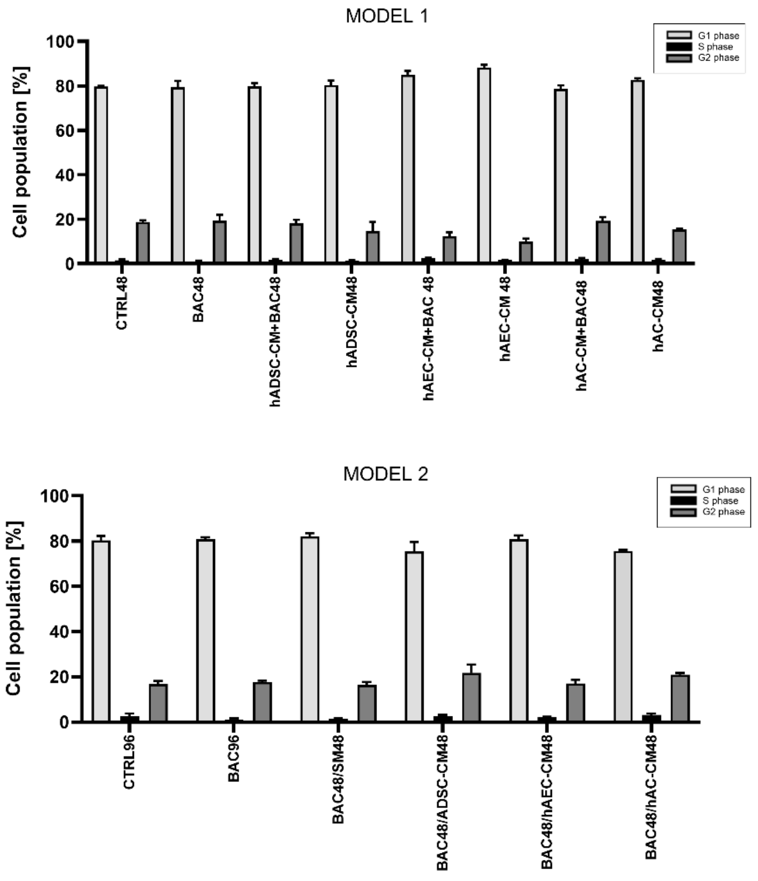

Supplementary Figure S4. Cell cycle distribution of LSCs in models 1 and 2 assessed by flow cytometry. Bar graphs show the percentage of cells in each phase. No significant differences in cell cycle phase distribution were observed between experimental groups.
